# Computational redesign of Fab CC12.3 with substantially better predicted binding affinity to SARS-CoV-2 than human ACE2 receptor

**DOI:** 10.1038/s41598-021-00684-x

**Published:** 2021-11-12

**Authors:** Wantanee Treewattanawong, Thassanai Sitthiyotha, Surasak Chunsrivirot

**Affiliations:** 1 Structural and Computational Biology Research Unit, Department of Biochemistry, Faculty of Science, Chulalongkorn University, Pathumwan, Bangkok, 10330 Thailand; 2 Department of Biochemistry, Faculty of Science, Chulalongkorn University, Pathumwan, Bangkok, 10330 Thailand

**Keywords:** Protein design, Antibody fragment therapy

## Abstract

SARS-CoV-2 is responsible for COVID-19 pandemic, causing large numbers of cases and deaths. It initiates entry into human cells by binding to the peptidase domain of angiotensin-converting enzyme 2 (ACE2) receptor via its receptor binding domain of S1 subunit of spike protein (SARS-CoV-2-RBD). Employing neutralizing antibodies to prevent binding between SARS-CoV-2-RBD and ACE2 is an effective COVID-19 therapeutic solution. Previous studies found that CC12.3 is a highly potent neutralizing antibody that was isolated from a SARS-CoV-2 infected patient, and its Fab fragment (Fab CC12.3) bound to SARS-CoV-2-RBD with comparable binding affinity to ACE2. To enhance its binding affinity, we employed computational protein design to redesign all CDRs of Fab CC12.3 and molecular dynamics (MD) to validate their predicted binding affinities by the MM-GBSA method. MD results show that the predicted binding affinities of the three best designed Fabs CC12.3 (CC12.3-D02, CC12.3-D05, and CC12.3-D08) are better than those of Fab CC12.3 and ACE2. Additionally, our results suggest that enhanced binding affinities of CC12.3-D02, CC12.3-D05, and CC12.3-D08 are caused by increased SARS-CoV-2-RBD binding interactions of CDRs L1 and L3. This study redesigned neutralizing antibodies with better predicted binding affinities to SARS-CoV-2-RBD than Fab CC12.3 and ACE2. They are promising candidates as neutralizing antibodies against SARS-CoV-2.

## Introduction

The ongoing coronavirus disease 2019 (COVID-19) pandemic caused by severe acute respiratory syndrome coronavirus 2 (SARS-CoV-2) has caused large numbers of morbidity and mortality^1–5^. SARS-CoV-2 has four main structural proteins including the nucleocapsid (N), membrane (M), envelope (E), and spike (S) protein^3,6–9^. Its spike protein consists of S1 and S2 subunits that are responsible for receptor recognition, viral attachment, and entry into human cells^10–13^. The receptor-binding domain (RBD) of the S1 subunit interacts with the peptidase domain (PD) of the angiotensin-converting enzyme 2 (ACE2) receptor, while the S2 subunit plays an important role in membrane fusion^3,4,7,14,15^. RBD of SARS-CoV-2 (SARS-CoV-2-RBD) is mainly recognized by the α1-helix with a minor contribution from the α2-helix and the linker between the β3 and β4 antiparallel strands of the ACE2 peptidase domain (ACE2-PD)^7,15^.

Disrupting the protein–protein interactions of SARS-CoV-2-RBD and ACE2-PD to prevent the entry of SARS-CoV-2 into human cells is a promising therapeutic strategy. Various potential therapeutic solutions such as neutralizing antibodies, small-molecule drugs, and peptide inhibitors have been widely investigated, and they can be used to prevent ACE2/SARS-CoV-2-RBD binding^1,16–22^. Neutralizing antibodies is an effective therapeutic solution for COVID-19 as they can effectively inhibit viral infection of human cells by blocking the binding interactions between SARS-CoV-2-RBD and ACE2-PD. Furthermore, some neutralizing antibodies such as sotrovimab^23,24^, REGEN-COV (casirivimab and imdevimab)^25–27^ and the combination of bamlanivimab and etesevimab^26,28^ have already been given an emergency use authorization from the U.S. Food and Drug Administration to treat mild-to-moderate COVID-19 in adults and pediatric patients.

The previous experimental study found that the Fab fragment of CC12.3 (Fab CC12.3), which was isolated from a SARS-CoV-2 infected patient, was among the top four highly potent neutralizing antibodies (IC_50_ of ~ 20 ng/ml) in a list of antibodies assayed against live replicating SARS-CoV-2 virus and pseudovirus^29^. Moreover, Fab CC12.3 bound to SARS-CoV-2-RBD with *K*_*d*_ of 14 nM^30^, which is comparable to *K*_*d*_ of ACE2 binding to SARS-CoV-2-RBD (14.7 nM)^31^, and it bound to an epitope that overlaps with ACE2-binding site. However, the binding affinity of Fab CC12.3 can be further enhanced to improve its effectiveness in preventing the SARS-CoV-2-RBD binding to human ACE2 receptor using computational techniques.

Computational protein design and molecular dynamics (MD) have been employed to develop potential therapeutic solutions for COVID-19 such as peptide inhibitors and antibodies. Recently, we employed computational protein design (Rosetta) and MD (AMBER) to design 25 mer-peptide binders (SPB25) of SARS-CoV-2-RBD with better predicted binding affinity than 23-mer peptide binder (SBP1)^32^, which is the experimentally proven 23-mer peptide binder of SARS-CoV-2-RBD, and human ACE2 receptor^33^. In terms of antibodies, Jiao Chen et al. performed virtual scanning mutageneses and MD to improve the binding affinity to SARS-CoV-2-RBD of P2B-2F6, which was isolated from single B cells of SARS-CoV-2 infected patients. They found that two P2B-2F6 mutants (H:V106R and H:V106R/H:P107Y) have higher binding affinities to SARS-CoV-2-RBD than P2B-2F6 and other mutants, suggesting that these two mutants might have higher neutralizing activity against SARS-CoV-2 than P2B-2F6^8^. Additionally, Mauricio Aguilar Rangel et al. developed a fragment-based computational design approach to design antibodies targeting epitopes on three antigens, including SARS-CoV-2-RBD. Their experimental results show that all designed antibodies are highly stable and bound to their targets with nanomolar affinities^34^.

The objective of this work is to significantly enhance the binding affinity of Fab CC12.3 to SARS-CoV-2-RBD, employing computational protein design (RosettaAntibodyDesign; RAbD) and MD (AMBER). We redesigned all complementarity-determining regions (CDRs) H1, H2, H3, L1, L2 and L3 of Fab CC12.3 so that its overall binding affinity to SARS-CoV-2-RBD is better than Fab CC12.3 and ACE2. The designed Fabs CC12.3 with increased predicted binding affinities to SARS-CoV-2-RBD are promising candidates that could potentially be used as neutralizing antibodies in preventing the binding of SARS-CoV-2-RBD and human ACE2 receptor.

## Results

### Computational design of Fab CC12.3

The crystal structure of Fab CC12.3/SARS-CoV-2-RBD complex (PDB code: 6XC4)^30^ was used as a designed template. Employing RAbD^35^ in Rosetta, CDRs H1, H2 and H3 of the heavy chain and CDRs L1, L2 and L3 of the light chain of Fab CC12.3 were redesigned to enhance the binding affinity of Fab CC12.3 to SARS-CoV-2-RBD so that its binding affinity is better than ACE2 and Fab CC12.3. CDR H3 was also redesigned with various chain lengths using GraftDesign^35^, and each residue of all CDRs was allowed to be any of standard amino acids. As shown in Table 1, nine designed Fabs CC12.3 (CC12.3-D01 to CC12.3-D09) with ΔG_bind (Rosetta)_ better than − 40.0 REU were chosen for MD simulations to validate whether their predicted binding affinities by the more accurate molecular mechanics–generalized born surface area (MM-GBSA) method^36–38^ (ΔG_bind (MM-GBSA)_) were better than that of Fab CC12.3 (ΔΔG_bind (MM-GBSA)_ < 0 kcal/mol).

**Table 1:** Predicted binding free energies (ΔG_bind (Rosetta)_) of designed Fabs CC12.3 to SARS-CoV-2-RBD and their CDR sequences.

System	ΔG_bind (Rosetta)_ (REU)	Number of residues	Heavy chain			Light chain		
			CDR H1 (23–35)	CDR H2 (50–58)	CDR H3 (93–102)	CDR L1 (24–34)	CDR L2 (49–56)	CDR L3 (89–97)
CC12.3	–	636	AASGFTVSSNYMS	VIYSGGSTF	ARDFGDFYFDY	RASQSVSSYLA	YGASSRAT	QQYGSSPRT
CC12.3-D01	− 250.7	639	KVSGFILSNAYMA	AIWTSGTTF	ATSIGGD**TRI**PGGS	RASEDIGYWLA	YDTSKLAS	QQYGEFPPT
CC12.3-D02	− 119.6	636	KTSGFTVSNTAMA	AIDASGSQY	ARLDYGSAFDY	KASQDIGYWLA	YDGSKLAE	LQNAEFPPT
CC12.3-D03	− 111.3	636	IVSGFDLSATGMS	IIYPSGTQF	ARQYTGSYFDY	RASEDIGHNIA	YDGSILAP	LQYAKFPPT
CC12.3-D04	− 101.1	632	VTSGFNLSASYMA	IIYTSGTTF	VTGL----FDY	RASEDIGHNIA	YNGSILAP	MQFAKFPPT
CC12.3-D05	− 82.3	632	KTSGFNLSATDMS	TIWASGTTF	VQE----GYIY	RASTDIGYFIA	YNTSQLAD	QQFGEFPPT
CC12.3-D06	− 74.0	634	KASGFDLSAAWMH	AIWASGVTY	ARG--LEVINL	KASTDIGTNIA	YDGSKLAP	QQGAEFPPT
CC12.3-D07	− 44.3	632	KTSGFNLSMTYMA	VIYASGTTF	VTGL----FDY	RASEDIGTNLA	YDGSKLAP	LQYAKHPPT
CC12.3-D08	− 43.4	630	QVSGFTLSASWMA	IIWASGTTF	TR------MDY	KASEDIGYWMA	YDTSKLAP	GQYTKLPLT
CC12.3-D09	− 43.3	630	KVSGFNLSATYMA	VIYASGTTY	AR------SGY	RASEDIGNFLA	YDGTKLAP	LQYGKLPRT

The mutated, inserted and deleted residues are underlined, highlighted in bold and represented with a hyphen, respectively.

### Validation by MD simulations

The MM-GBSA method was employed to calculate ΔG_bind (MM-GBSA)_ to determine whether the designed Fab CC12.3 have better predicted binding affinity than Fab CC12.3. MD simulations were performed on the structures of Fab CC12.3 and the nine designed Fabs CC12.3 in complex with SARS-CoV-2-RBD. The RMSD values of all atoms and backbone atoms were calculated to analyze structural stabilities of all systems. Figure S1 shows that all systems are likely to be stable in the range of 80–100 ns based on their RMSD values; therefore, these trajectories were used for further analyses. The values of ΔG_bind (MM-GBSA)_ of all systems during the 80–100 ns trajectories were calculated to predict the binding affinities of all systems. As shown in Table 2, the values of ΔG_bind (MM-GBSA)_ of Fab CC12.3/SARS-CoV-2-RBD complex is − 72.5 ± 0.3 kcal/mol. Three of nine designed Fabs CC12.3 such as CC12.3-D02, CC12.3-D05, and CC12.3-D08 have better ΔG_bind (MM-GBSA)_ than CC12.3 with ΔΔG_bind (MM-GBSA)_ of − 6.1 ± 0.4, − 1.6 ± 0.4, and − 24.0 ± 0.5 kcal/mol, respectively. Moreover, the predicted binding affinities of CC12.3, CC12.3-D02 (ΔG_bind (MM-GBSA)_ = − 78.6 ± 0.3 kcal/mol), CC12.3-D05 (ΔG_bind (MM-GBSA)_ = − 74.1 ± 0.3 kcal/mol), and CC12.3-D08 (ΔG_bind (MM-GBSA)_ = − 96.5 ± 0.4 kcal/mol) are also better than that of ACE2 (ΔG_bind (MM-GBSA)_ = − 71.2 ± 0.4 kcal/mol)^32^.

**Table 2:** The binding free energies of Fab CC12.3 and designed Fabs CC12.3 binding to SARS-CoV-2-RBD, as calculated by Rosetta and MM-GBSA method.

System	ΔG_bind (Rosetta)_ (REU)	ΔG_bind (MM-GBSA)_ (kcal/mol)	ΔΔG_bind (MM-GBSA)_ (kcal/mol)
ACE2^32^	–	− 71.2 ± 0.4	1.3 ± 0.5
CC12.3	–	− 72.5 ± 0.3	0.0 ± 0.4
CC12.3-D01	− 250.7	− 67.1 ± 0.6	5.4 ± 0.7
CC12.3-D02	− 119.6	− 78.6 ± 0.3	− 6.1 ± 0.4
CC12.3-D03	− 111.3	− 63.7 ± 0.4	8.8 ± 0.5
CC12.3-D04	− 101.1	− 66.4 ± 0.3	6.1 ± 0.4
CC12.3-D05	− 82.3	− 74.1 ± 0.3	− 1.6 ± 0.4
CC12.3-D06	− 74.0	− 69.9 ± 0.8	2.6 ± 0.9
CC12.3-D07	− 44.3	− 66.8 ± 0.4	5.7 ± 0.5
CC12.3-D08	− 43.4	− 96.5 ± 0.4	− 24.0 ± 0.5
CC12.3-D09	− 43.3	− 55.9 ± 0.4	16.6 ± 0.5

**Figure 1:**
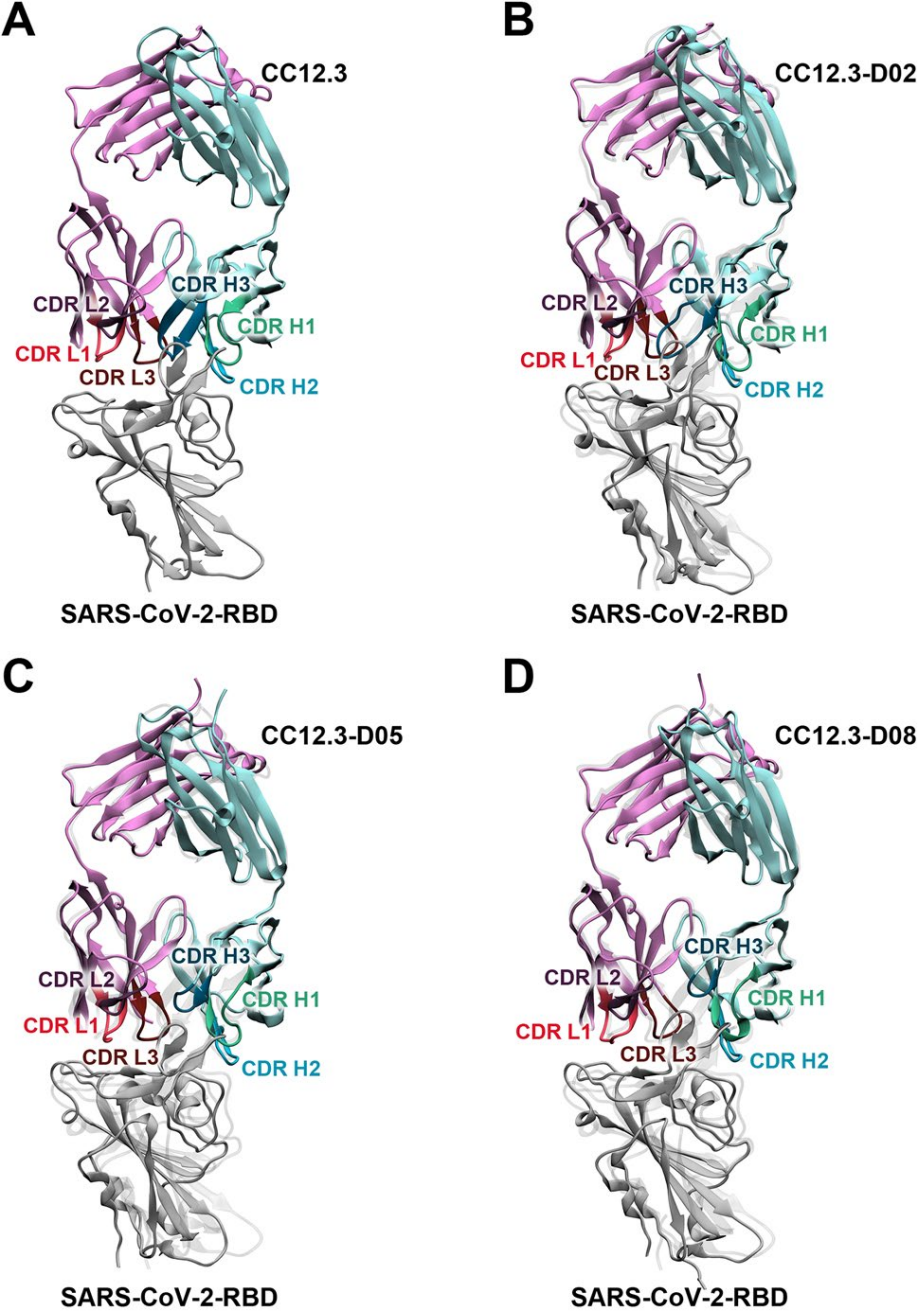
Overall structures of the heavy chain (light blue) and light chain (purple) of (**A**) CC12.3, (**B**) CC12.3-D02, (**C**) CC12.3-D05, and (**D**) CC12.3-D08 binding to SARS-CoV-2-RBD (gray). CDRs H1, H2, H3, L1, L2, and L3 are colored in green, blue, dark green, dark pink, dark purple, and dark red, respectively. The designed Fabs CC12.3/SARS-CoV-2-RBD were superimposed with Fab CC12.3/SARS-CoV-2-RBD (light gray).

The structures of Fab CC12.3 and the three best designed Fabs CC12.3 binding to SARS-CoV-2-RBD with better predicted binding affinities than Fab CC12.3 and ACE2 are illustrated in Fig. 1. Overall, the binding positions and orientations of CC12.3-D02, CC12.3-D05, and CC12.3-D08 to SARS-CoV-2-RBD are relatively similar to that of Fab CC12.3. However, CDR H3 in the heavy chain of Fab CC12.3 was redesigned to have various chain lengths. As a result, the chain lengths of CDRs H3 in CC12.3-D05 (7 residues) and CC12.3-D08 (5 residues) are shorter than that of CC12.3 (11 residues), while the chain length of CDR H3 in CC12.3-D02 is similar to that of CC12.3 (Table S1).

**Figure 2:**
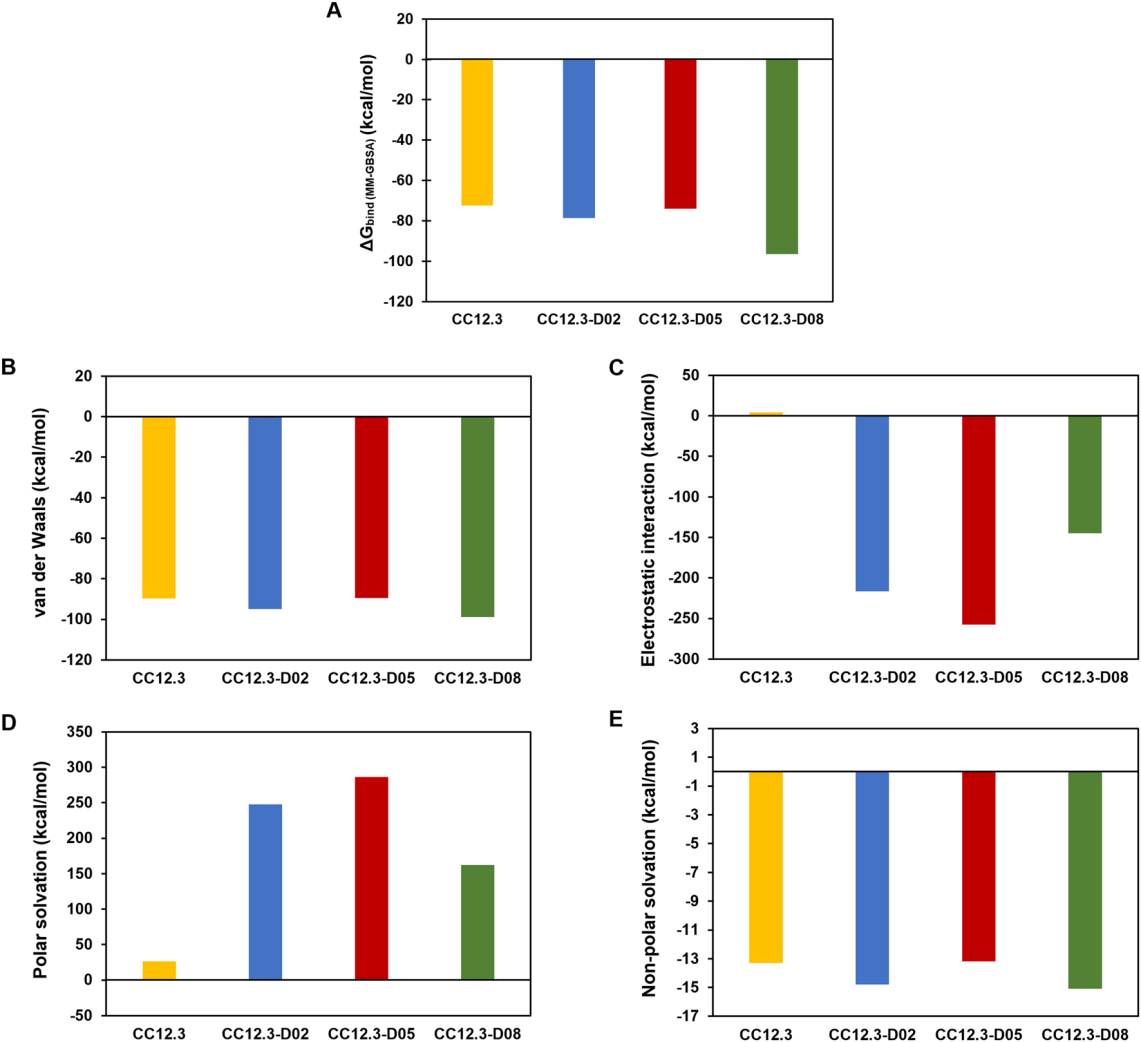
Binding free energy components of CC12.3/SARS-CoV-2-RBD, CC12.3-D02/SARS-CoV-2-RBD, CC12.3-D05/SARS-CoV-2-RBD, and CC12.3-D08/SARS-CoV-2-RBD. (**A**) ΔG_bind (MM-GBSA)_, (**B**) van der Waals energy, (**C**) electrostatic interaction, (**D**) polar solvation, and (**E**) non-polar solvation.

In terms of binding free energy components of designed Fabs CC12.3 (Fig. 2), the electrostatic interaction terms are the main components contributing to the favorable predicted binding affinities of CC12.3-D02, CC12.3-D05, and CC12.3-D08 to SARS-CoV-2-RBD, while the electrostatic interaction term of Fab CC12.3 has unfavorable contribution to the predicted binding affinity. The van der Waals energy and non-polar solvation terms of Fab CC12.3, CC12.3-D02, CC12.3-D05, and CC12.3-D08 have favorable contributions to the predicted binding affinities to SARS-CoV-2-RBD. However, the polar solvation terms contribute unfavorably to the predicted binding affinity.

CC12.3-D08 is the designed Fab CC12.3 with the best predicted binding affinity with the ΔG_bind (MM-GBSA)_ value of − 96.5 ± 0.4 kcal/mol. Its predicted binding affinity is better than that of CC12.3 with the ΔΔG_bind (MM-GBSA)_ value of − 24.0 ± 0.5 kcal/mol. The favorable binding affinity of CC12.3-D08 to SARS-CoV-2-RBD is caused by the substantial increase in the favorable electrostatic interaction term as well as the increase in the favorable van der Waals energy and non-polar solvation terms, as compared to that of CC12.3. However, the unfavorable polar solvation term of CC12.3-D08 is worse than that of CC12.3. The predicted binding affinities of CC12.3-D02 and CC12.3-D05 are also better than that of CC12.3. The favorable binding affinity of CC12.3-D02 and CC12.3-D05 to SARS-CoV-2-RBD is mostly caused by the substantial increase in the favorable electrostatic interaction terms. The favorable van der Waals energy and non-polar solvation terms of CC12.3-D02 are also better than that of CC12.3, while the favorable van der Waals energy and non-polar solvation terms of CC12.3-D05 are similar to those of CC12.3. The unfavorable polar solvation terms of these three designed Fabs CC12.3 are worse than that of CC12.3.

### Identification of important binding residues

**Figure 3:**
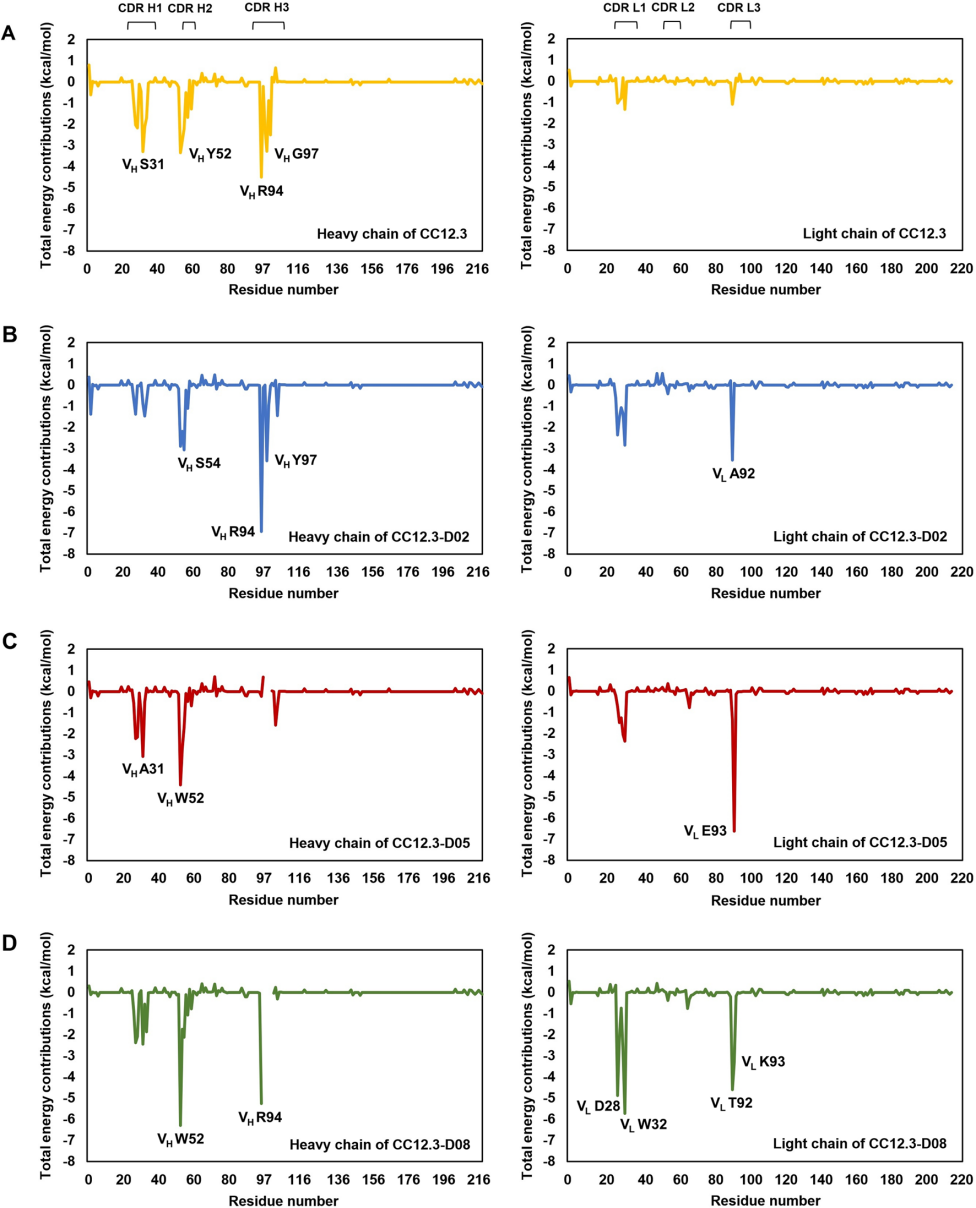
Per-residue free energy decomposition of (**A**) CC12.3, (**B**) CC12.3-D02, (**C**) CC12.3-D05, and (**D**) CC12.3-D08 in binding to SARS-CoV-2-RBD. The left and right panels show per-residue free energy decomposition of residues in CDR H1, H2 and H3, and CDR L1, L2 and L3, respectively. Residues with high binding affinities that have the total energy contribution better than − 3 kcal/mol are labelled.

To identify important binding residues of Fab CC12.3 and designed Fabs CC12.3 to SARS-CoV-2-RBD, per residue free energy decomposition was computed and shown in Fig. 3. In this study, a residue with the total energy contribution better than − 1.0 kcal/mol was defined to be an important binding residue. Furthermore, a residue with the total energy contribution better than − 3.0 kcal/mol was defined to be a residue with high binding affinity. Focusing on residues in CDRs of CC12.3, V_H_ residues F27, T28, S31, N32 and Y33 in CDR H1, V_H_ residues Y52, S53, G54, S56 and F58 in CDR H2, V_H_ residues R94, F96, G97 and F99 in CDR H3, V_L_ residues S28 and Y32 in CDR L1, and V_L_ G92 in CDR L3 were predicted to be the important binding residues to SARS-CoV-2-RBD. Additionally, V_H_ residues S31 (CDR H1), Y52 (CDR H2), R94 (CDR H3), and G97 (CDR H3) were predicted to have high binding affinity to SARS-CoV-2-RBD.

In terms of per-residue free energy decomposition of CC12.3-D02, the important binding residues are V2 of the heavy chain, V_H_ residues F27 and T32 in CDR H1, V_H_ residues D52, A53, S54 and S56 in CDR H2, V_H_ residues R94, Y97 and Y102 in CDR H3, V_L_ residues D28, I29, G30, Y31 and W32 in CDR L1, and V_L_ A92 in CDR L3. V_H_ residues S54 (CDR H2), R94 (CDR H3) and Y97 (CDR H3), and V_L_ A92 (CDR L3) were also predicted to have high binding affinities. Furthermore, the total energy contributions of the mutated residues such as V_H_ residues S54 and Y97, and V_L_ residues D28, I29, G30, Y31, W32 and A92 were favorably increased from − 2.2, − 3.3, − 1.0, − 0.9, − 0.8, 0.2, − 1.3 and − 1.1 kcal/mol in CC12.3 to − 3.1, − 3.6, − 2.4, − 1.5, − 1.1, − 1.4, − 2.9 and − 3.6 kcal/mol in CC12.3-D02, respectively. Additionally, the total energy contributions of other residues such as V2, V_H_ R94 and V_H_ Y102 were also substantially increased from − 0.6, − 4.5 and − 0.0 kcal/mol in CC12.3 to − 1.4, − 6.9 and − 1.4 kcal/mol in CC12.3-D02, respectively.

In terms of per-residue free energy decomposition of CC12.3-D05, V_H_ residues F27, N28 and A31 in CDR H1, V_H_ residues W52, A53 and S54 in CDR H2, V_H_ I101 in CDR H3, V_L_ residues I29, G30, Y31 and F32 in CDR L1, and V_L_ residues G92 and E93 in CDR L3 were predicted to be important binding residues to SARS-CoV-2-RBD. V_H_ A31, V_H_ W52, and V_L_ E93 were also predicted to be the residues with high binding affinity of CC12.3-D05. The mutated residues such as V_H_ residues W52 and I101 as well as V_L_ residues I29, G30, Y31, F32 and E93 were predicted to favorably increase the total energy contribution from − 3.3, 0.7, − 0.9, − 0.8, 0.2, − 1.3 and − 0.5 kcal/mol in CC12.3 to − 4.4, − 1.6, − 1.5, − 1.3, − 2.1, − 2.4 and − 6.6 kcal/mol in CC12.3-D05. Moreover, the total energy contributions of residues V_H_ F27 and V_L_ G92 were also increased from − 2.0 and − 1.1 kcal/mol in CC12.3 to − 2.2 and − 1.4 kcal/mol in CC12.3-D05, respectively.

The important binding residues of CC12.3-D08 to SARS-CoV-2-RBD are V_H_ residues F27, T28, A31 and W33 in CDR H1, V_H_ residues W52, A53, S54 and T56 in CDR H2, V_H_ R94 in CDR H3, V_L_ residues D28, I29, Y31 and W32 in CDR L1, and V_L_ residues T92 and K93 in CDR L3. V_H_ W52 was predicted to have the highest binding affinity to SARS-CoV-2-RBD, followed by V_L_ W32, V_H_ R94, V_L_ D28, V_L_ T92, and V_L_ K93, respectively. Additionally, the total energy contributions of the mutated residues such as V_H_ W52, V_L_ residues D28, I29, Y31, W32, T92 and K93 were favorably increased from − 3.3, − 1.0, − 0.9, 0.2, − 1.3, − 1.1 and − 0.5 kcal/mol in CC12.3 to − 6.3, − 4.9, − 2.1, − 2.8, − 5.7, − 4.6 and − 3.3 kcal/mol in CC12.3-D08. In addition, the total energy contributions of V_H_ F27 and V_H_ R94 were also favorably increased from − 2.0 and − 4.5 kcal/mol in CC12.3 to − 2.4 and − 5.3 kcal/mol in CC12.3-D08, respectively.

### Hydrogen bond and pi interactions

**Figure 4:**
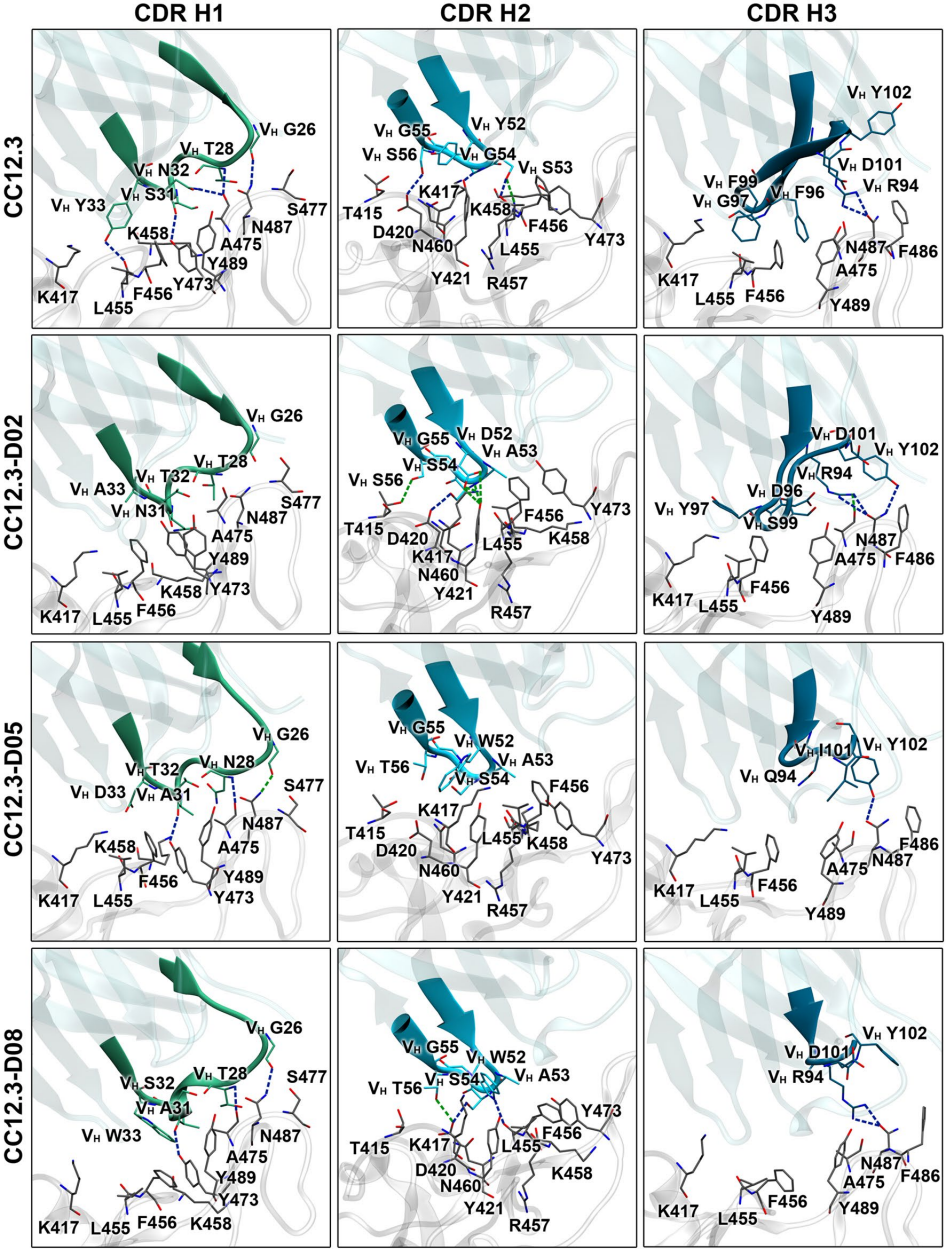
Key binding interactions between SARS-CoV-2-RBD and heavy chains of CC12.3, CC12.3-D02, CC12.3-D05, and CC12.3-D08. Strong and medium hydrogen bonds are shown in blue and green dashed lines, respectively.

**Figure 5:**
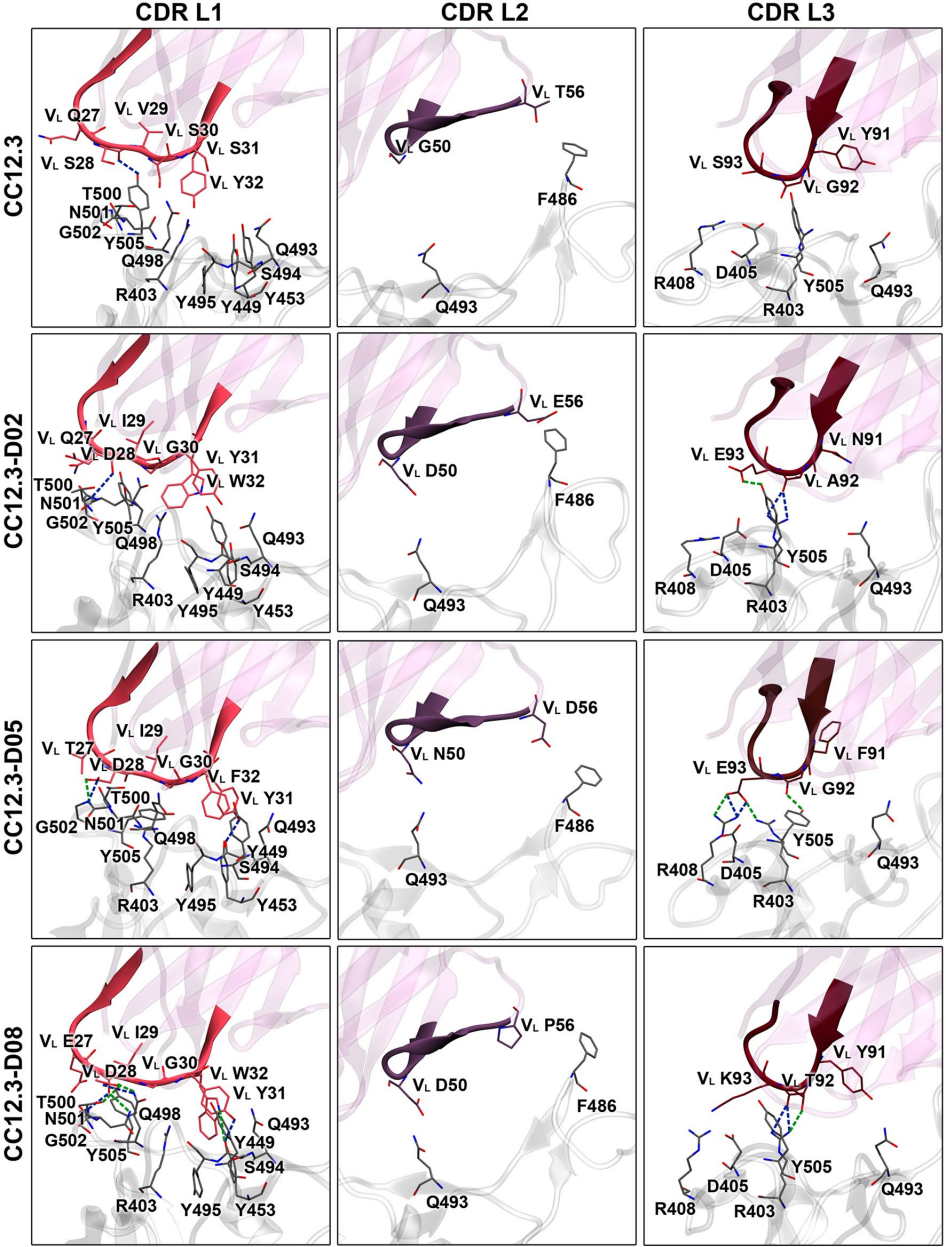
Key binding interactions between SARS-CoV-2-RBD and light chains of CC12.3, CC12.3-D02, CC12.3-D05, and CC12.3-D08. Strong and medium hydrogen bonds are shown in blue and green dashed lines, respectively.

To identify important hydrogen bond and pi interactions of all systems, hydrogen bond occupations (Tables 3 and S2–S5), pi–pi, cation–pi, anion–pi, sigma–pi, and alkyl–pi interactions (Tables 4 and S6) were analyzed. The key binding interactions of Fab CC12.3 and designed Fabs CC12.3 to SARS-CoV-2-RBD are shown in Figs. 4 and 5. Overall, the total numbers of predicted hydrogen bonds and pi interactions of CC12.3-D02, CC12.3-D05 and CC12.3-D08 are more than those of CC12.3, supporting the binding energy result that these designed CC12.3 were predicted to bind better to SARS-CoV-2-RBD than CC12.3.

**Table 3:** Numbers of hydrogen bonds of CC12.3, CC12.3-D02, CC12.3-D05, and CC12.3-D08 involved in SARS-CoV-2-RBD binding.

System	Number of hydrogen bonds					Residue that forms a hydrogen bond with SARS-CoV-2-RBD						
	Strong	Medium	Weak	Very weak	Total	Outside CDRs	Heavy chain			Light chain		
							CDR H1	CDR H2	CDR H3	CDR L1	CDR L2	CDR L3
CC12.3	11	1	4	13	29	–	G26 (s)	S53 (s,m)	R94 (s)	S28 (s)	–	G92 (vw)
							T28 (s)	G54 (s)	G97 (w)	S30 (vw)		S93 (vw)
							S31 (s,vw)	G55 (vw)				
							N32 (s)	S56 (s,w,vw)				
							Y33 (s)					
CC12.3-D02	7	7	5	21	40	Q1(H) (vw)	G26 (vw)	D52 (m,w,vw)	R94 (s,m,vw)	Q27 (vw)	–	A92 (s)
						S67(L) (vw)	N31 (w,vw)	S54 (s,m,w)	D96 (vw)	D28 (s,vw)		E93 (m,w)
							T32 (vw)	S56 (m,vw)	Y102 (s)	G30 (vw)		
							A33 (w)			Y31 (vw)		
CC12.3-D05	7	5	3	20	35	S67(L) (vw)	G26 (m)	A53 (vw)	Y102 (s,vw)	D28 (s,m,vw)	–	G92 (m,vw)
							N28 (s)	S54 (vw)		Y31 (s,vw)		E93 (s,m,w,vw)
							S30 (vw)	T56 (vw)				
							A31 (s)					
							D33 (vw)					
CC12.3-D08	13	6	5	18	42	S67(L) (s)	G26 (s)	W52 (s,vw)	R94 (s,vw)	E27 (vw)	D50 (w,vw)	Y91 (vw)
							T28 (s)	S54 (s,vw)		D28 (s,m,w)		T92 (s,m)
							A31 (s)	T56 (m,vw)		G30 (vw)		K93 (vw)
										Y31 (s,w,vw)		
										W32 (m,vw)		

*s* strong hydrogen bond, *m* medium hydrogen bond, *w* weak hydrogen bond, and *vw* very weak hydrogen bond.

**Table 4:** Number of pi interactions of CC12.3, CC12.3-D02, CC12.3-D05, and CC12.3-D08 involved in SARS-CoV-2-RBD binding**.**

System	Number of pi interactions					
	pi–pi	Cation–pi	Anion–pi	Sigma–pi	Alkyl–pi	Total
CC12.3	4	2	–	–	3	9
CC12.3-D02	4	2	1	1	5	13
CC12.3-D05	4	1	–	–	5	10
CC12.3-D08	8	3	–	2	4	17

In terms of hydrogen bond and pi interactions between Fab CC12.3 and SARS-CoV-2-RBD, V_H_ residues G26, S31 and Y33 in CDR H1 were predicted to form strong hydrogen bonds with N487, Y473 and L455 of SARS-CoV-2-RBD, respectively. V_H_ T28 and V_H_ N32 in CDR H1 were also predicted to form two strong hydrogen bonds with the backbone carbonyl of A475 of SARS-CoV-2-RBD. Additionally, V_H_ Y33 was predicted to form one pi–pi interaction with F456 of SARS-CoV-2-RBD. V_H_ S53 in CDR H2 was predicted to form strong and medium hydrogen bonds with R457 of SARS-CoV-2-RBD. V_H_ G54 and V_H_ S56 were also predicted to form strong hydrogen bonds with Y421 and D420 of SARS-CoV-2-RBD, respectively. Moreover, there is one predicted alkyl–pi interaction formed between V_H_ Y52 and K417 of SARS-CoV-2-RBD. Two strong hydrogen bonds were also predicted to form between V_H_ R94 in CDR H3 and N487 of SARS-CoV-2-RBD. Furthermore, there are two predicted pi–pi interactions (V_H_ F96⋯F456 and V_H_ F96⋯Y489), one predicted cation–pi interaction (V_H_ R94⋯F486) and one predicted alkyl–pi interaction (V_H_ F99⋯L455) formed between CDR H3 and SARS-CoV-2-RBD. In terms of hydrogen bond and pi interactions between CDRs L1, L2 and L3, and SARS-CoV-2-RBD, there are one predicted strong hydrogen bond (V_L_ S28⋯Y505), one predicted pi–pi interaction (V_L_ Y32⋯Y505), and one predicted cation–pi interaction (V_L_ Y32⋯R403) formed between CDR L1 and SARS-CoV-2-RBD. The CDR L2 and L3 were not predicted to form any strong hydrogen bonds, medium hydrogen bonds or pi-interactions with SARS-CoV-2-RBD. Moreover, Fab CC12.3 was predicted to additionally form 4 weak hydrogen bonds and 13 very weak hydrogen bonds with SARS-CoV-2-RBD. These hydrogen bonds were predicted to form between SARS-CoV-2-RBD and V_H_ S31, V_H_ G55, V_H_ S56, V_H_ G97, V_L_ S30, V_L_ G92 and V_L_ S93 of CC12.3. In addition, there is one alkyl–pi interaction formed between V2 in the heavy chain of Fab CC12.3 and SARS-CoV-2-RBD.

For the hydrogen bond and pi interactions of CC12.3-02, the mutated residue V_H_ A33 in CDR H1 was predicted to form one alkyl–pi interaction with F456 of SARS-CoV-2-RBD. However, CDR H1 of CC12.3-D02 was not predicted to form any strong or medium hydrogen bonds. The mutated residue V_H_ S54 in CDR H2 was predicted to form strong and medium hydrogen bonds with D420 and Y421 of SARS-CoV-2-RBD, respectively. Three medium hydrogen bonds were also predicted to form between the mutated residue V_H_ D52 with K417 and Y421 of SARS-CoV-2-RBD. V_H_ S56 in CDR H2 was additionally predicted to form one medium hydrogen bond with T415 of SARS-CoV-2-RBD. Moreover, the mutated residue V_H_ A53 in CDR H2 was predicted to form two alkyl–pi interactions with F456 and Y473 of SARS-CoV-2-RBD. V_H_ R94 and V_H_ Y102 of the CDR H3 were predicted to form three strong hydrogen bonds and one medium hydrogen bond with N487 and A475 of SARS-CoV-2-RBD. Additionally, the mutated residue V_H_ Y97 in CDR H3 of CC12.3-D02 was predicted to form pi–pi, cation–pi and alkyl–pi interactions with F456, K417 and L455 of SARS-CoV-2-RBD, respectively. Additionally, V_H_ Y102 in CDR H3 was also predicted to form one pi–pi interaction with F486 of SARS-CoV-2-RBD. In terms of hydrogen bond and pi-interactions of CDRs L1, L2 and L3 of CC12.3-D02, strong hydrogen bond was predicted to form between the mutated residue V_L_ D28 in CDR L1 and G502 of SARS-CoV-2-RBD. The mutated residues V_L_ Y31 and V_L_ W32 in CDR L1 were also predicted to form two pi–pi, one cation–pi and one sigma–pi interactions with R403, Y449 and Y505 of SARS-CoV-2-RBD. Although CDR L2 was not predicted to form any hydrogen bonds, the mutated residue V_L_ E56 in CDR L2 was predicted to form one anion–pi interaction with F486 of SARS-CoV-2-RBD. The mutated residues V_L_ A92 and V_L_ E93 in CDR L3 were predicted to form two strong hydrogen bonds and one medium hydrogen bond with R403 and Y505 of SARS-CoV-2-RBD. Moreover, there is one predicted alkyl–pi interaction formed between the mutated residue V_L_ A92 and Y505 of SARS-CoV-2-RBD. Furthermore, other residues including the mutated residues of CC12.3-D02 were also predicted to form 5 weak hydrogen bonds and 21 very weak hydrogen bonds with SARS-CoV-2-RBD.

In terms of the hydrogen bond and pi-interactions between CC12.3-D05 and SARS-CoV-2-RBD, the backbones of the mutated residues V_H_ N28 and V_H_ A31 in CDR H1 were predicted to form two strong hydrogen bonds with A475 and Y473 of SARS-CoV-2-RBD, respectively. One medium hydrogen bond was also predicted to form between V_H_ G26 in CDR H1 and N487 of SARS-CoV-2-RBD. Moreover, there is one alkyl–pi interaction between the mutated residue V_H_ A31 in CDR H1 and Y473 of SARS-CoV-2-RBD. CDR H2 was not predicted to form any strong or medium hydrogen bonds. However, the mutated residue V_H_ W52 in CDR H2 was predicted to form pi–pi, cation–pi and alkyl–pi interactions with K417 and Y421 of SARS-CoV-2-RBD. Additionally, V_H_ Y102 in CDR H3 was predicted to form one strong hydrogen bond and one pi–pi interaction with N487 and F486 of SARS-CoV-2-RBD, respectively. The mutated residue of V_H_ I101 in CDR H3 was also predicted to form two alkyl–pi interactions with F486 and Y489 of SARS-CoV-2-RBD. For the hydrogen bond and pi interactions of CDR L1, L2 and L3 of CC12.3-05, the mutated residue V_L_ D28 in CDR L1 was predicted to form one strong hydrogen bond and one medium hydrogen bond with G502 of SARS-CoV-2-RBD. Furthermore, the mutated residue V_L_ Y31 in CDR L1 was also predicted to form one strong hydrogen bond and one pi–pi interaction with S494 and Y449 of SARS-CoV-2-RBD, respectively. One pi–pi interaction was predicted to form between the mutated residue V_L_ F32 in CDR L1 and Y505 of SARS-CoV-2-RBD. Moreover, the mutated residue V_L_ E93 in CDR L3 was predicted to form two strong hydrogen bonds and two medium hydrogen bonds with R403 and R408 of SARS-CoV-2-RBD. One medium hydrogen bond was also predicted to form between V_L_ G92 in CDR L3 and Y505 of SARS-CoV-2-RBD. However, CDR L2 was not predicted to form any hydrogen bonds with SARS-CoV-2-RBD, and no pi-interaction was predicted to form between CDR L2/L3 and SARS-CoV-2-RBD. Moreover, CC12.3-D05 was predicted to form 3 weak hydrogen bonds and 20 very weak hydrogen bonds between CDRs and SARS-CoV-2-RBD. Furthermore, V2 in the heavy chain of CC12.3-D05 was also predicted to form one alkyl–pi interaction with SARS-CoV-2-RBD.

The total numbers of hydrogen bonds and pi-interactions of CC12.3-D08 are more than that those CC12.3, CC12.3-D02 and CC12.3-D05, supporting the binding free energy result that it has better predicted binding affinity to SARS-CoV-2-RBD than CC12.3, CC12.3-D02 and CC12.3-D05. The mutated residue V_H_ A31 in CDR H1 was predicted to form one strong hydrogen bond with Y473 of SARS-CoV-2-RBD. Other residues such as V_H_ G26 and V_H_ T28 were also predicted to form strong hydrogen bonds with N487 and A475 of SARS-CoV-2-RBD, respectively. There are two pi–pi interactions (V_H_ W33⋯F456 and V_H_ W33⋯Y489) and one alkyl–pi interaction (V_H_ A31⋯Y473) formed between these mutated residues in CDR H1 and SARS-CoV-2-RBD. The mutated residue V_H_ W52 and V_H_ S54 in CDR H2 were also predicted to form two strong hydrogen bonds with L455 and D420 of SARS-CoV-2-RBD, respectively. One medium hydrogen bond was predicted to form between the mutated residue V_H_ T56 and D420 of SARS-CoV-2-RBD. Additionally, CDR H2 of CC12.3-D08 was predicted to form two pi–pi interactions (V_H_ W52⋯Y421 and V_H_ W52⋯F456) between the mutated residue V_H_ W52 and SARS-CoV-2-RBD. The mutated residue V_H_ W52 was also predicted to form one cation–pi, two sigma–pi and one alkyl–pi interactions with K417 of SARS-CoV-2-RBD. Moreover, two strong hydrogen bonds were predicted to form between V_H_ R94 in CDR H3 and N487 of SARS-CoV-2-RBD. V_H_ Y102 and V_H_ R94 were also predicted to form pi–pi and cation–pi interactions with F486 of SARS-CoV-2-RBD. In terms of the hydrogen bonds and pi-interactions of CDR L1, L2 and L3, the mutated residue V_L_ D28 in CDR L1 was predicted to form two strong hydrogen bonds and three medium hydrogen bonds with Q498, T500, N501 and G502 of SARS-CoV-2-RBD. Additionally, the mutated residues V_L_ Y31 and V_L_ W32 in CDR L1 were also predicted to form one strong hydrogen bond and one medium hydrogen bond with S494 of SARS-CoV-2-RBD. CDR L1 of CC12.3-D08 was predicted to form three pi–pi interactions (V_L_ Y31⋯Y449, V_L_ W32⋯Y453 and V_L_ W32⋯Y495), one cation–pi interaction (V_L_ W32⋯R403) and one alkyl–pi interaction (V_L_ I29⋯Y505) between these mutated residues and SARS-CoV-2-RBD. In addition, the mutated residue V_L_ T92 in CDR L3 was predicted to form two strong hydrogen bonds and one medium hydrogen bond with R403 of SARS-CoV-2-RBD. Additionally, there is one alkyl–pi interaction formed between the mutated residue V_L_ K93 of CDR L3 and Y505 of SARS-CoV-2-RBD. However, CDR L2 was not predicted to form any strong hydrogen bonds, medium hydrogen bonds or pi-interactions with SARS-CoV-2-RBD. Furthermore, S67 in the light chain of CC12.3-D08 was also predicted to form one strong hydrogen bond with Q498 of SARS-CoV-2-RBD. Furthermore, 5 weak hydrogen bonds and 18 very weak hydrogen bonds were additionally predicted to form between CDRs and SARS-CoV-2-RBD.

## Discussion

Caused by SARS-CoV-2, COVID-19 pandemic is responsible for large numbers of global cases and deaths. SARS-CoV-2 initiates entry into human cells by binding to ACE2-PD though the SARS-CoV-2-RBD of its spike protein. Therefore, disrupting the binding between SARS-CoV-2-RBD and ACE2-PD to prevent virus entry is one of effective therapeutic solutions for COVID-19. SARS-CoV-2-RBD-targeting antibodies (neutralizing antibodies) can be used to neutralize SARS-CoV-2 by blocking ACE2-PD binding, and some antibodies such as sotrovimab^23,24^, the combination of casirivimab and imdevimab^25–27^, and the combination of bamlanivimab and etesevimab^26,28^ have already been given an emergency use authorization from the U.S. Food and Drug Administration. The previous experimental study found that Fab CC12.3, which was isolated from a SARS-CoV-2 infected patient and was specific for SARS-CoV-2-RBD^29^, bound to SARS-CoV-2-RBD with *K*_d_ of 14 nM^30^, which is comparable to *K*_d_ of ACE2 binding to SARS-CoV-2-RBD (14.7 nM)^31^, and it also binds to SARS-CoV-2-RBD at a binding site similar to ACE2. Among antibodies assayed against live replicating SARS-CoV-2 virus and pseudovirus, CC12.3 was also among the top four highly potent neutralizing antibodies^29^. However, the binding affinity of Fab CC12.3 to SARS-CoV-2-RBD can be further enhanced using computational techniques to improve its effectiveness in preventing the binding between SARS-CoV-2-RBD and ACE2.

To further increase the binding affinity of Fab CC12.3, this study employed computational antibody design (RosettaAntibodyDesign) and MD (AMBER) to redesign all CDRs (CDR H1, H2, H3, L1, L2 and L3) of Fab CC12.3 so that their predicted binding affinities to SARS-CoV-2-RBD are substantially better than ACE2 and Fab CC12.3. The chain length of CDR H3 in the heavy chain of Fab CC12.3 was additionally allowed to be varied. After computational protein design, the total of nine designed Fabs CC12.3 with ΔG_bind (Rosetta)_ better than − 40.0 REU were obtained from RosettaAntibodyDesign, and they were chosen for MD simulations to validate whether their predicted binding affinities by the more accurate MM-GBSA method (ΔG_bind (MM-GBSA)_) were better than that of Fab CC12.3 and ACE2.

MD results show that the predicted binding affinity to SARS-CoV-2-RBD of Fab CC12.3 (− 72.5 ± 0.3 kcal/mol) is comparable to that of ACE2 (− 71.2 ± 0.4 kcal/mol)^32^, and these results are in reasonable agreement with the experimental results that Fab CC12.3 bound to SARS-CoV-2-RBD (*K*_d_ = 14 nM)^30^ with comparable affinity to ACE2 (14.7 nM)^31^. Three best designed Fabs CC12.3 such as CC12.3-D02, CC12.3-D05 and CC12.3-D08 were predicted to bind to SARS-CoV-2-RBD better than Fab CC12.3 with ΔΔG_bind (MM-GBSA)_ of − 6.1 ± 0.4, − 1.6 ± 0.4, and − 24.0 ± 0.5 kcal/mol, respectively, and their predicted binding affinities are also better than ACE2. Our results suggest that they should be able to bind to SARS-CoV-2-RBD better than Fab CC12.3 and ACE2, experimentally. The ranking of the predicted binding affinities of Fab CC12.3, ACE2 and the three best designed Fabs CC12.3 to SARS-CoV-2-RBD (best to worst) is CC12.3-D08 > CC12.3-D02 > CC12.3-D05 > CC12.3 ≈ ACE2. Moreover, the binding positions and orientations of the three best designed Fabs CC12.3 to SARS-CoV-2-RBD are relatively similar to that of Fab CC12.3, which binds to SARS-CoV-2-RBD at a binding site similar to ACE2. These findings suggest that they should bind to SARS-CoV-2-RBD in a similar binding pose and could potentially disrupt the binding of ACE2 to SARS-CoV-2-RBD.

CC12.3-D08 is the most promising designed Fab CC12.3 because its predicted binding affinity is substantially better than ACE2 (by about 25 kcal/mol), CC12.3 (by about 24 kcal/mol), CC12.3-D02 and CC12.3-D05. This result is supported by the fact that its total numbers of predicted hydrogen bonds (involving V_H_ residues G26, T28, A31, W52, S54, T56, R94, V_L_ residues E27, D28, G30, Y31, W32, D50, Y91, T92, K93, and S67 of the light chain) and pi interactions (involving V_H_ residues A31, W33, W52, R94, Y102 and V_L_ residues I29, Y31, W32, K93) are higher than those of CC12.3, CC12.3-D02 and CC12.3-D05. Additionally, the predicted numbers of strong and medium hydrogen bonds (involving V_H_ residues G26, T28, A31, W52, S54, T56, R94, V_L_ residues D28, Y31, W32, T92, and S67 of the light chain) of CC12.3-D08 are higher than those of CC12.3, CC12.3-D02 and CC12.3-D05. The mutated residues of CC12.3-D08 were also predicted to form hydrogen bonds (involving V_H_ residues A31, W52, S54, T56, and V_L_ residues E27, D28, G30, Y31, W32, D50, T92, K93) and pi interactions (involving V_H_ residues A31, W33, W52, and V_L_ I29, Y31, W32, K93) with SARS-CoV-2-RBD. The per-residue free energy decomposition results suggest V_H_ residues F27, T28, A31, W33, W52, A53, S54, T56, R94, and V_L_ residues D28, I29, Y31, W32, T92, K93 as important binding residues. Moreover, CC12.3-D08 was predicted to cause substantial favorable increase in the total energy contribution of the mutated residues such as V_H_ W52, V_L_ residues D28, I29, Y31, W32, T92, K93, and the total energy contributions of other residues such as V_H_ F27 and V_H_ R94 as compared to that of CC12.3. Overall, the enhanced binding affinity of CC12.3-D08 to SARS-CoV-2-RBD is mostly caused by the increase in the binding interactions of the light chain, especially CDR L1. This finding is supported by the fact that the total numbers of hydrogen bonds (involving V_L_ residues E27, D28, G30, Y31 and W32) and pi-interactions (involving V_L_ residues I29, Y31, W32) in CDR L1 of CC12.3-D08 are higher than those of CC12.3, CC12.3-D02 and CC12.3-D05. Moreover, the CDR L2 (V_L_ D50) was also predicted to form weak and very weak hydrogen bonds with SARS-CoV-2-RBD, while the CDR L2 of CC12.3, CC12.3-D02 and CC12.3-D05 were not predicted to form any hydrogen bonds. Additionally, the total numbers of predicted hydrogen bonds (involving V_L_ residues Y91, T92, and K93) and pi-interactions (involving V_L_ K93) of CDR L3 of CC12.3-D08 are higher than those of CC12.3. Furthermore, S67 in the light chain of CC12.3-D08 was also predicted to form strong hydrogen bond with SARS-CoV-2-RBD. In terms of the binding interactions to SARS-CoV-2-RBD in the heavy chain of CC12.3-D08, the binding interactions of CDR H3 of CC12.3-D08 are worse than those of CC12.3 probably because its chain length is shorter than that of CC12.3. This result is supported by the fact that the total numbers of predicted hydrogen bonds (involving V_H_ R94) and pi-interactions (involving V_H_ R94 and V_H_ Y102) of CDR H3 of CC12.3-D08 are lower than those of CC12.3. Moreover, the total numbers of predicted hydrogen bonds of CDR H1 and H2 of CC12.3-D08 are lower than those of CC12.3. However, the total numbers of predicted pi-interactions of CDR H1 and H2 of CC12.3-D08 are higher than those of CC12.3. In any case, since the epitopes of CDR H3 are relatively flat, have only a small pocket for CDR H3 insertion and cannot accommodate long CDR H3, short CDR H3 of CC12.3-D08 should be able to bind to these epitopes^30^.

The predicted binding affinity of CC12.3-D02 is better than those of CC12.3 and CC12.3-D05. This result is supported by the fact that its total numbers of predicted hydrogen bonds (involving V_H_ residues G26, N31, T32, A33, D52, S54, S56, R94, D96, Y102, V_L_ residues Q27, D28, G30, Y31, A92, E93, Q1 of the heavy chain, and S67 of the light chain) and pi interactions (involving V_H_ residues A33, A53, Y97, Y102, and V_L_ residues Y31, W32, E56, A92) of CC12.3-D02 are higher than those of CC12.3 and CC12.3-D05, and the total numbers of predicted strong and medium hydrogen bonds (involving V_H_ residues D52, S54, S56, R94, Y102, and V_L_ residues D28, A92, E93) of CC12.3-D02 are higher than those of CC12.3 and CC12.3-D05. The results from per-residue free energy decomposition suggest V_H_ residues F27, T32, D52, A53, S54, S56, R94, Y97, Y102, and V_L_ residues D28, I29, G30, Y31, W32, A92, and V2 of the heavy chain as important binding residues. Furthermore, CC12.3-D02 was predicted to cause the increase in the total energy contribution of the mutated residues such as V_H_ S54, Y97, V_L_ residues D28, I29, G30, Y31, W32, A92, and the total energy contributions of other residues such as V_H_ R94, V_H_ Y102, and V2 of the heavy chain as compared to that of CC12.3. Overall, the fact that CC12.3-D02 has better predicted binding affinity to SARSCoV-2-RBD than CC12.3 is mostly caused by the increase in the total numbers of predicted hydrogen bonds of CDR L1 (involving V_L_ Q27, D28, G30 and Y31) and CDR L3 (involving V_L_ A92 and E93) of CC12.3-D02 as compared to those of CC12.3. Additionally, CDRs L1, L2 and L3 were predicted to have increased total numbers of pi-interaction (involving V_L_ Y31, W32, E56 and A92) between CC12.3-D02 and SARS-CoV-2-RBD. Moreover, CDR H3 of CC12.3-D02, whose chain length is similar to that of CC12.3, was also predicted to contribute to the enhanced binding affinity of CC12.3-D02 caused by the increase in the total numbers of predicted hydrogen bonds (involving V_H_ R94, D96 and Y102) with SARS-CoV-2-RBD.

CC12.3-D05 was predicted to bind better to SARS-CoV2-RBD than CC12.3. This result is supported by the fact that the total numbers of predicted hydrogen bonds (involving V_H_ residues G26, N28, S30, A31, D33, A53, S54, T56, Y102, V_L_ residues D28, Y31, G92, E93, and S67 of the light chain) and pi interactions (involving V_H_ residues A31, W52, I101, Y102, V_L_ residues Y31, F32, and V2 of the heavy chain) of CC12.3-D05 are higher than that of CC12.3. The total numbers of strong and medium hydrogen bonds (involving V_H_ G26, N28, A31, Y102, and V_L_ D28, Y31, G92, E93) are also higher than those of CC12.3. Furthermore, the results from per-residue free energy decomposition suggest V_H_ residues F27, N28, A31, W52, A53, S54, and I101, V_L_ residues I29, G30, Y31, F32, G92 and E93 as important binding residues. Additionally, CC12.3-D05 was predicted to have increased total energy contribution of the mutated residues such as V_H_ residues W52, I101, and V_L_ residues I29, G30, Y31, F32, E93, and other residues such as V_H_ F27 and V_L_ G92 as compared to those of CC12.3. However, CC12.3-D05 has the worst predicted binding affinity among the three best designed Fabs CC12.3, and this result is supported by the fact that its total numbers of predicted hydrogen bonds and pi interactions are the lowest among the three best designed Fabs CC12.3. Overall, the enhanced binding affinity of CC12.3-D05 is mostly caused by the increase in the total numbers of predicted hydrogen bonds of CDR L1 (involving V_L_ D28 and Y31) and CDR L3 (involving V_L_ G92 and E93) of CC12.3-D05 as compared to those of CC12.3. By contrast, the binding interactions in the heavy chain of CC12.3-D05 are worse than those of CC12.3. This result is supported by the fact that the total numbers of predicted hydrogen bonds (involving V_H_ residues G26, N28, S30, A31, D33 in CDR H1, and V_H_ residues A53, S54, T56 in CDR H2) and pi interactions (involving V_H_ A31 in CDR H1, and V_H_ W52 in CDR H2) of the heavy chain of CC12.3-D05 are lower than that of CC12.3. Similar to CC12.3-D08, the chain length of CDR H3 of CC12.3-D05 is shorter than that of CC12.3. As a result, the binding interactions of CDR H3 of CC12.3-D05 are worse than those of CC12.3 as supported by the decrease in the total numbers of predicted hydrogen and pi interactions of CDR H3 of CC12.3-D05 with SARS-CoV-2-RBD, as compared to CC12.3.

Previous study found that the light chain of Fab CC12.3 formed small interactions with SARS-CoV-2-RBD^30^. However, the light chains of our best designed Fabs CC12.3 were redesigned to form more favorable interactions with SARS-CoV-2-RBD than those of Fab CC12.3. Based on the binding interaction analyses of the three best designed Fabs CC12.3 and Fab CC12.3, our findings suggest that the enhanced binding affinities of CC12.3-D02, CC12.3-D05 and DD12.3-D08 are mostly caused by the increased binding interactions of the light chain (CDR L1 and L3) as compared to those of CC12.3. Therefore, our results suggest CDR L1 and L3 as promising design targets of CC12.3 to further increase its binding affinity to SARS-CoV-2-RBD.

The ranking of the predicted binding affinities of designed Fabs CC12.3 to SARS-CoV-2-RBD could be validated experimentally by performing binding kinetics experiments using biolayer interferometry as described in detail in the work by Yuan et al.^30^. After expression and purification, the binding kinetics of ACE2, Fab CC12.3 and designed Fabs CC12.3 to SARS-CoV-2-RBD can be measured using five concentrations at twofold dilution ranging from 500 to 31.25 nM^30^. Then, the K_d_ values of ACE2, Fab CC12.3 and designed Fabs CC12.3 can be obtained from the curve fitting, and the ranking of K_d_ values can be used to validate the ranking of the predicted binding affinities.

In conclusion, we used computational protein design and MD to design neutralizing antibodies, using Fab CC12.3 as a template, with better predicted binding affinities to SARS-CoV-2-RBD than Fab CC12.3 and human ACE2 receptor. CC12.3-D02, CC12.3-D05 and CC12.3-D08 are the best designed Fabs CC12.3 with better predicted binding affinities to SARS-CoV-2-RBD, as calculated by the MM-GBSA method, than Fab CC12.3 and human ACE2 receptor. CC12.3-D08 is the best designed Fab CC12.3 with substantially better predicted binding affinities to SARS-CoV-2-RBD than CC12.3 and ACE2 by about 24 and 25 kcal/mol, respectively. The increased binding interactions of CDR L1 and L3 are most likely responsible for the enhanced binding affinities of CC12.3-D02, CC12.3-D05 and CC12.3-D08, supporting CDR L1 and L3 as promising design targets for further enhancing the binding affinity of CC12.3. CC12.3-D02, CC12.3-D05 and CC12.3-D08 are promising candidates that could potentially be used as neutralizing antibodies to prevent the binding of SARS-CoV-2-RBD and ACE2.

## Methods

### Structure preparation

The crystal structure of SARS-CoV-2-RBD bound to Fab CC12.3 complex was obtained from the protein data bank (PDB code: 6XC4)^30^. H++ server^39^ was employed to protonate all ionized amino acids at the physiological pH 7.4. The LEaP module of AMBER18^40^ was used to build the final structure of Fab CC12.3/SARS-CoV-2-RBD complex.

### Computational protein design

The structure of Fab CC12.3/SARS-CoV-2-RBD complex was used as a design template to increase the binding affinity between Fab CC12.3 and SARS-CoV-2-RBD. The RosettaAntibodyDesign (RAbD)^35^ in RosettaDesign module of Rosetta3.12^41^ was employed to design the CDR H1, H2 and H3 of the heavy chain and the CDR L1, L2 and L3 of the light chain of Fab CC12.3. RAbD requires the Rosetta Antibody Design Database that can be obtained from PyIgClassify (http://dunbrack2.fccc.edu/pyigclassify) for CDR structural classifications of CDR H1, H2, H3, L1, L2 and L3. The RAbD protocol consists of outer and inner Monte Carlo cycles. For each outer cycle, GraftDesign was used to design the CDR H3 of the heavy chain with various chain lengths by randomly choosing a CDR from the canonical cluster database. In the inner cycle, each CDR residue was allowed to be any of standard amino acids using SequenceDesign (SeqDesign), and their structures were energetically minimized. 500 independent runs were performed, and the total of 500 conformations of designed sequences were obtained. The binding free energy (ΔG_bind (Rosetta)_) of each designed conformation was calculated in Rosetta Energy Unit (REU). The designed sequences/conformations with ΔG_bind (Rosetta)_ better than − 40.0 REU were chosen for MD simulations.

### MD simulations and analyses

The LEaP module of AMBER18^40^ was employed to immerse the complex structures of Fab CC12.3/SARS-CoV-2-RBD and designed Fabs CC12.3/SARS-CoV-2-RBD in isomeric truncated octahedral TIP3P water boxes with the buffer distance of 13 Å, using protein ff14SB^42^ and GLYCAM06j-1^43^ force field parameters. Then, the five steps minimization procedure was applied to reduced unfavorable interactions of each complex^32,33,44–56^. All steps included 2500 steps of steepest-descent and 2500 steps of conjugated gradient with different restrains on proteins. In the first step, the heavy atoms of protein were restrained with a force constant of 10 kcal/(mol Å), while the hydrogen atoms and water molecules were minimized. The force constants of 10, 5 and 1 kcal/(mol Å) were subsequently used to restrain the backbone of protein in the second, third and fourth steps of minimizations, respectively. In the last step, the whole system was minimized with no restrain. After minimization, all systems were simulated under the periodic boundary condition, using the GPU (CUDA) version of PMEMD module^57–59^. The SHAKE algorithm^60^ was employed to constrain all bonds relating to hydrogen atoms, allowing 0.002 ps time steps simulations. To control the simulation temperature, the Langevin dynamic technique^61^ was used with a collision frequency of 1 ps^−1^. All systems were heated from 0 to 310 K (physiological temperature) for 200 ps in the NVT ensemble, while the protein backbones were restrained with the force constant of 10 kcal/(mol Å). All systems were then equilibrated at 310 K for 300 ps in the NVT ensemble with no restraint. Finally, all systems were simulated in the NPT ensemble at 310 K and 1 atm for 100 ns.

In terms of analyses, the root mean square deviation (RMSD) values were computed to analyze the structural stability of each system. The last 20 ns trajectories of all systems with stable RMSD values were selected for further analyses. The molecular mechanics–generalized born surface area (MM-GBSA) method^36–38^ was employed to calculate the total binding free energies (ΔG_bind (MM-GBSA)_) of all systems to predict the binding affinity between Fab CC12.3/designed Fabs CC12.3 and SARS-CoV-2-RBD. The designed Fabs CC12.3 with better predicted binding affinity than Fab CC12.3 were further analyzed in terms of decomposition of free energy per residue and binding interactions. Decomposition of free energy per residue was computed to identify important binding residues between Fab CC12.3/designed Fabs CC12.3 and SARS-CoV-2-RBD. A residue with the total energy contribution better than − 1.0 kcal/mol was defined to be an important binding residue, and a residue with the total energy contribution better than − 3.0 kcal/mol was defined to be a residue with high binding affinity. To determine the interactions that are crucial for increased binding affinities of the designed Fabs CC12.3, hydrogen bond and pi interactions were analyzed. A hydrogen bond was considered to occur if the following criteria were met: (1) a proton donor–acceptor distance ≤ 3.5 Å and (2) a donor–H–acceptor bond angle ≥ 120°^46–48,51^. The strengths of hydrogen bond interactions were classified into four levels: (1) strong hydrogen bonds (hydrogen bond > 75%), (2) medium hydrogen bonds (75% ≥ hydrogen bond > 50%), (3) weak hydrogen bonds (50% ≥ hydrogen bond > 25%) and (4) very weak hydrogen bonds (25% ≥ hydrogen bond > 5%)^46,48,49^.

## Supplementary Information

Supplementary Information.

## References

[1] Huang X, Pearce R, Zhang Y (2020). *De**novo* design of protein peptides to block association of the SARS-CoV-2 spike protein with human ACE2. Aging.

[2] Hui DS (2020). The continuing 2019-nCoV epidemic threat of novel coronaviruses to global health—The latest 2019 novel coronavirus outbreak in Wuhan, China. Int. J. Infect. Dis..

[3] Adem KA, Shanti A, Stefanini C, Lee S (2020). Inhibition of SARS-CoV-2 entry into host cells using small molecules. Pharmaceuticals.

[4] Kar S, Leszczynski J (2020). From animal to human: Interspecies analysis provides a novel way of ascertaining and fighting COVID-19. Innovation.

[5] Matheson NJ, Lehner PJ (2020). How does SARS-CoV-2 cause COVID-19?. Science.

[6] Li G (2020). Coronavirus infections and immune responses. J. Med. Virol..

[7] Tai W (2020). Characterization of the receptor-binding domain (RBD) of 2019 novel coronavirus: Implication for development of RBD protein as a viral attachment inhibitor and vaccine. Cell. Mol. Immunol..

[8] Chen J (2021). Rational optimization of a human neutralizing antibody of SARS-CoV-2. Comput. Biol. Med..

[9] Ortega JT, Serrano ML, Pujol FH, Rangel HR (2020). Role of changes in SARS-CoV-2 spike protein in the interaction with the human ACE2 receptor: An *in**silico* analysis. EXCLI J..

[10] Raghuvamsi PV (2021). SARS-CoV-2 S protein: ACE2 interaction reveals novel allosteric targets. Elife.

[11] Lu R (2020). Genomic characterisation and epidemiology of 2019 novel coronavirus: Implications for virus origins and receptor binding. Lancet.

[12] Li F (2016). Structure, function, and evolution of coronavirus spike proteins. Annu. Rev. Virol..

[13] Bosch BJ, van der Zee R, de Haan CAM, Rottier PJM (2003). The coronavirus spike protein is a class I virus fusion protein: Structural and functional characterization of the fusion core complex. J. Virol..

[14] Coutard B (2020). The spike glycoprotein of the new coronavirus 2019-nCoV contains a furin-like cleavage site absent in CoV of the same clade. Antiviral.

[15] Yan R (2020). Structural basis for the recognition of SARS-CoV-2 by full-length human ACE2. Science.

[16] Li Z (2020). Development and clinical application of a rapid IgM-IgG combined antibody test for SARS-CoV-2 infection diagnosis. J. Med. Virol..

[17] Monteil V (2020). Inhibition of SARS-CoV-2 infections in engineered human tissues using clinical-grade soluble human ACE2. Cell.

[18] Yuan M (2020). A highly conserved cryptic epitope in the receptor binding domains of SARS-CoV-2 and SARS-CoV. Science.

[19] Wu Y (2020). A noncompeting pair of human neutralizing antibodies block COVID-19 virus binding to its receptor ACE2. Science.

[20] Cao L (2020). De novo design of picomolar SARS-CoV-2 miniprotein inhibitors. Science.

[21] Han Y, Kraĺ P (2020). Computational design of ACE2-based peptide inhibitors of SARS-CoV-2. ACS Nano.

[22] Singh R, Bhardwaj VK, Sharma J, Kumar D, Purohit R (2021). Identification of potential plant bioactive as SARS-CoV-2 spike protein and human ACE2 fusion inhibitors. Comput. Biol. Med..

[23] Gupta A (2021). Early Covid-19 treatment with SARS-CoV-2 neutralizing antibody sotrovimab. medRxiv.

[24] U.S. Food and Drug Administration, Coronavirus (COVID-19) Update: FDA Authorizes Additional Monoclonal Antibody for Treatment of COVID-19. *FDA Official Website*. https://www.fda.gov/news-events/press-announcements/coronavirus-covid-19-update-fda-authorizes-additional-monoclonal-antibody-treatment-covid-19 (2021).

[25] Tuccori M (2020). Anti-SARS-CoV-2 neutralizing monoclonal antibodies: Clinical pipeline. MAbs.

[26] Almehdi AM (2021). SARS-CoV-2 spike protein: Pathogenesis, vaccines, and potential therapies. Infection.

[27] U.S. Food and Drug Administration, Coronavirus (COVID-19) Update: FDA Authorizes Monoclonal Antibodies for Treatment of COVID-19. *FDA Official Website*. https://www.fda.gov/news-events/press-announcements/coronavirus-covid-19-update-fda-authorizes-monoclonal-antibodies-treatment-covid-19 (2020).

[28] U.S. Food and Drug Administration, Coronavirus (COVID-19) Update: FDA Revokes Emergency Use Authorization for Monoclonal Antibody Bamlanivimab. *FDA Official Website*. https://www.fda.gov/news-events/press-announcements/coronavirus-covid-19-update-fda-revokes-emergency-use-authorization-monoclonal-antibody-bamlanivimab (2021).

[29] Rogers TF (2020). Isolation of potent SARS-CoV-2 neutralizing antibodies and protection from disease in a small animal model. Science.

[30] Yuan M (2020). Structural basis of a shared antibody response to SARS-CoV-2. Science.

[31] Wrapp D (2020). Cryo-EM structure of the 2019-nCoV spike in the prefusion conformation. Science.

[32] Sitthiyotha T, Chunsrivirot S (2020). Computational design of 25-mer peptide binders of SARS-CoV-2. J. Phys. Chem. B.

[33] Sitthiyotha T, Chunsrivirot S (2021). Computational design of SARS-CoV-2 peptide binders with better predicted binding affinities than human ACE2 receptor. Sci. Rep..

[34] Rangel MA (2021). Fragment-based computational design of antibodies targeting structured epitopes. bioRxiv.

[35] Adolf-Bryfogle J (2018). RosettaAntibodyDesign (RAbD): A general framework for computational antibody design. PLoS Comput. Biol..

[36] Miller BR (2021). MMPBSA.py: An efficient program for end-state free energy calculations. J. Chem. Theory Comput..

[37] Ylilauri M, Pentikäinen OT (2013). MMGBSA as a tool to understand the binding affinities of filamin–peptide interactions. J. Chem. Inf. Model..

[38] Genheden S, Ryde U (2015). The MM/PBSA and MM/GBSA methods to estimate ligand-binding affinities. Expert Opin. Drug Discov..

[39] Gordon JC (2005). H++: A server for estimating p*K*_a_s and adding missing hydrogens to macromolecules. Nucleic Acids Res..

[40] Case D (2018). AMBER 18.

[41] Leaver-Fay A (2011). ROSETTA3: An object-oriented software suite for the simulation and design of macromolecules. Methods Enzymol..

[42] Maier JA (2015). f14SB: Improving the accuracy of protein side chain and backbone parameters from ff99SB. J. Chem. Theory Comput..

[43] Kirschner KN (2008). GLYCAM06: A generalizable biomolecular force field. Carbohydrates. J. Comput. Chem..

[44] Mokmak W, Chunsrivirot S, Assawamakin A, Choowongkomon K, Tongsima S (2013). Molecular dynamics simulations reveal structural instability of human trypsin inhibitor upon D50E and Y54H mutations. J. Mol. Model..

[45] Mokmak W (2014). Molecular dynamics of interactions between rigid and flexible antifolates and dihydrofolate reductase from pyrimethamine-sensitive and pyrimethamine-resistant *Plasmodium**falciparum*. Chem. Biol. Drug Des..

[46] Sitthiyotha T, Pichyangkura R, Chunsrivirot S (2018). Molecular dynamics provides insight into how N251A and N251Y mutations in the active site of *Bacillus**licheniformis* RN-01 levansucrase disrupt production of long-chain levan. PLoS ONE.

[47] Kanjanatanin P (2019). Computational design of *Bacillus**licheniformis* RN-01 levansucrase for control of the chain length of levan-type fructooligosaccharides. Int. J. Biol. Macromol..

[48] Punnatin P, Chanchao C, Chunsrivirot S (2020). Molecular dynamics reveals insight into how N226P and H227Y mutations affect maltose binding in the active site of α-glucosidase II from European honeybee, *Apis**mellifera*. PLoS ONE.

[49] Klaewkla M, Pichyangkura R, Charoenwongpaiboon T, Wangpaiboon K, Chunsrivirot S (2020). Computational design of oligosaccharide producing levansucrase from *Bacillus**licheniformis* RN-01 to improve its thermostability for production of levan-type fructooligosaccharides from sucrose. Int. J. Biol. Macromol..

[50] Na Ayutthaya PP, Chanchao C, Chunsrivirot S (2018). Insight into the substrate specificity change caused by the Y227H mutation of α-glucosidase III from the European honeybee (*Apis**mellifera*) through molecular dynamics simulations. PLoS ONE.

[51] Charoenwongpaiboon T (2019). Modulation of fructooligosaccharide chain length and insight into the product binding motif of *Lactobacillus**reuteri* 121 inulosucrase. Carbohydr. Polym..

[52] Charoenwongpaiboon T (2019). Rational re-design of *Lactobacillus**reuteri* 121 inulosucrase for product chain length control. RSC Adv..

[53] Charoenwongpaiboon T (2020). Conserved calcium-binding residues at the Ca-I site involved in fructooligosaccharide synthesis by *Lactobacillus**reuteri* 121 inulosucrase. ACS Omega.

[54] Manissorn J (2020). Biochemical and structural investigation of GnnA in the lipopolysaccharide biosynthesis pathway of *Acidithiobacillus**ferrooxidans*. ACS Chem. Biol..

[55] Wangpaiboon K, Sitthiyotha T, Chunsrivirot S, Charoenwongpaiboon T, Pichyangkura R (2021). Unravelling regioselectivity of *Leuconostoc**citreum* ABK-1 alternansucrase by acceptor site engineering. Int. J. Mol. Sci..

[56] Klaewkla M, Pichyangkura R, Charoenwongpaiboon T, Wangpaiboon K, Chunsrivirot S (2020). Computational Design of Oligosaccharide-Producing Levansucrase from *Bacillus**licheniformis* RN-01 to increase its stability at high temperature. Int. J. Biol. Macromol..

[57] Götz AW (2012). Routine Microsecond molecular dynamics simulations with AMBER on GPUs. 1. Generalized born. J. Chem. Theory Comput..

[58] Le Grand S, Götz AW, Walker RC (2013). SPFP: Speed without compromise—A mixed precision model for GPU accelerated molecular dynamics simulations. Comput. Phys. Commun..

[59] Salomon-Ferrer R, Götz AW, Poole D, Le Grand S, Walker RC (2013). Routine microsecond molecular dynamics simulations with AMBER on GPUs. 2. Explicit solvent particle mesh Ewald. J. Chem. Teory Comput..

[60] York DM, Darden TA, Pedersen LG (1993). The effect of long-range electrostatic interactions in simulations of macromolecular crystals: A comparison of the Ewald and truncated list methods. J. Chem. Phys..

[61] Wu X, Brooks BR (2003). Self-guided Langevin dynamics simulation method. Chem. Phys. Lett..

