# Supplementary material for: Computational redesign of Fab CC12.3 with substantially better predicted binding affinity to SARS-CoV-2 than human ACE2 receptor
Source: Sci Rep. 2021 Nov 12;11:22202. doi: 10.1038/s41598-021-00684-x (PMC8590000; doi:10.1038/s41598-021-00684-x)
Supplement: Supplementary file 1 — Supplementary Information. [file 41598_2021_684_MOESM1_ESM.pdf]

# Computational redesign of Fab CC12.3 with substantially better predicted binding affinity to SARS-CoV-2 than human ACE2 receptor

*Wantanee Treewattanawong[a], Thassanai Sitthiyotha[a], Surasak Chunsrivirot[a,b,*]*

*[a]Structural and Computational Biology Research Unit, Department of Biochemistry, Faculty of Science, Chulalongkorn University, Pathumwan, Bangkok 10330, Thailand.*

*[b]Department of Biochemistry, Faculty of Science, Chulalongkorn University, Pathumwan, Bangkok 10330, Thailand.*

*[*]Surasak Chunsrivirot*
*Email: surasak.ch@chula.ac.th*

**Contents**

**Figure S1.** RMSD plots of Fab CC12.3 and designed Fabs CC12.3 in complex with SARS-CoV-2-RBD, S2

**Table S1.** Residues in CDR H3 of Fabs CC12.3, CC12.3-D02, CC12.3-D05, and CC12.3-D08, S3

**Table S2.** Hydrogen bond occupations of Fab CC12.3 involved in SARS-CoV-2-RBD binding, S4

**Table S3.** Hydrogen bond occupations of Fab CC12.3-D02 involved in SARS-CoV-2-RBD binding, S5

**Table S4.** Hydrogen bond occupations of Fab CC12.3-D05 involved in SARS-CoV-2-RBD binding, S6

**Table S5.** Hydrogen bond occupations of Fab CC12.3-D08 involved in SARS-CoV-2-RBD binding, S7

**Table S6.** Pi interactions of Fabs CC12.3, CC12.3-D02, CC12.3-D05, and CC12.3-D08 involved in SARS-CoV-2-RBD binding, S8

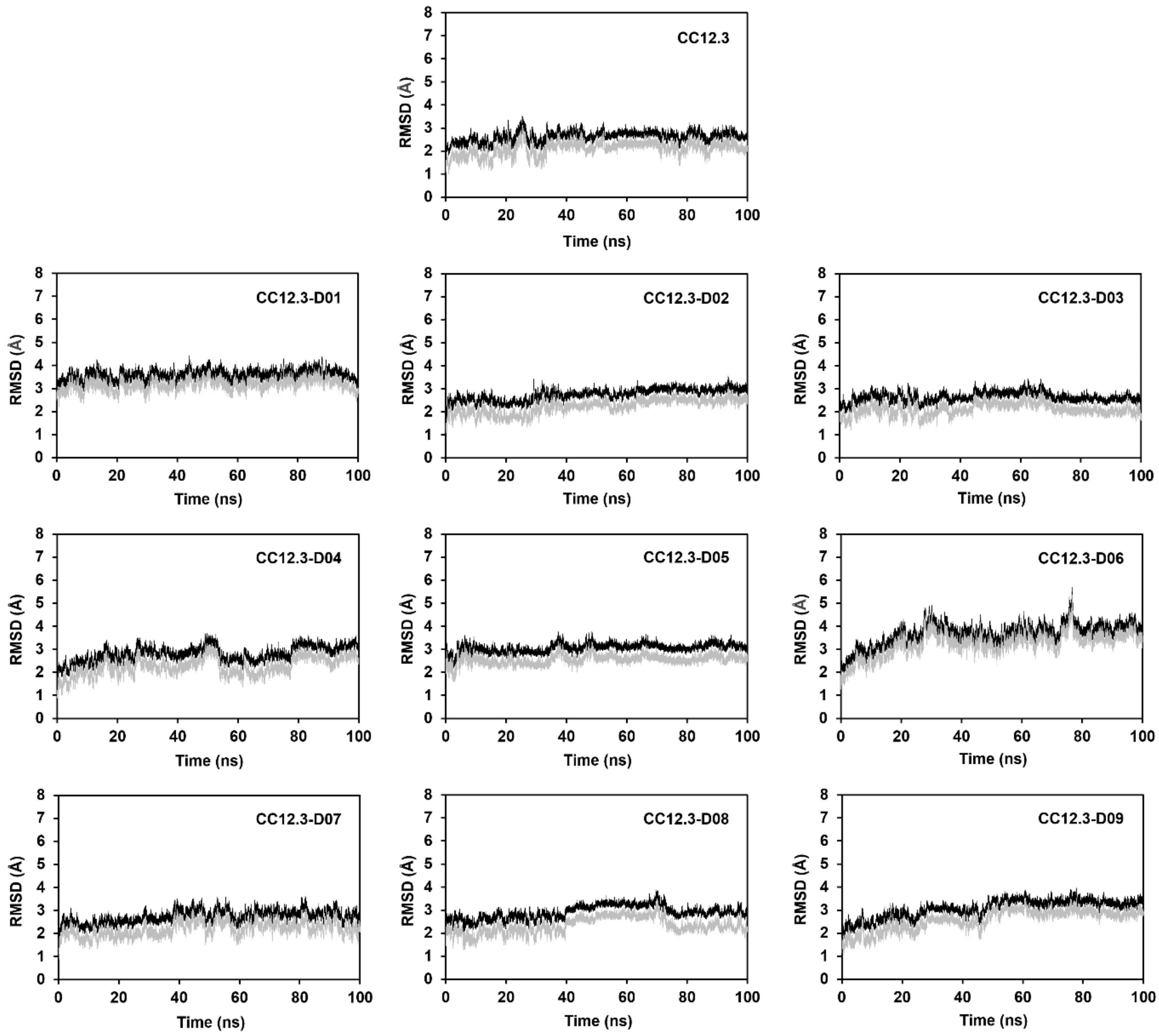


**Figure S1.** RMSD plots of Fab CC12.3 and designed Fabs CC12.3 in complex with SARS-CoV-2-RBD. The RMSD values of all atoms and backbone atoms are shown in black and gray respectively.

**Table S1.** Residues in CDR H3 of Fabs CC12.3, CC12.3-D02, CC12.3-D05, and CC12.3-D08.

| system | number of residues | Residues | | | | | | | | | | |
|---|---|---|---|---|---|---|---|---|---|---|---|---|
| | | 93 | 94 | 95 | 96 | 97 | 98 | 99 | 100 | 100A | 101 | 102 |
| CC12.3 | 636 | A | R | D | F | G | D | F | Y | F | D | Y |
| CC12.3-D02 | 636 | A | R | L | D | Y | G | S | A | F | D | Y |
| CC12.3-D05 | 632 | V | Q | E | | | | | G | Y | I | Y |
| CC12.3-D08 | 630 | T | R | | | | | | | M | D | Y |

**Table S2.** Hydrogen bond occupations of Fab CC12.3 involved in SARS-CoV-2-RBD binding.

| CDRs | Acceptor | DonorH | Donor | Hydrogen bond occupancy (%) |
|---|---|---|---|---|
| H1 | G26@O | N487@HD21 | N487@ND2 | 90.8 |
| | A475@O | T28@H | T28@N | 85.8 |
| | S31@O | Y473@HH | Y473@OH | 99.3 |
| | S31@OG | K458@HZ1 | K458@NZ | 5.6 |
| | S31@OG | K458@HZ2 | K458@NZ | 6.5 |
| | S31@OG | K458@HZ3 | K458@NZ | 6.2 |
| | A475@O | N32@HD21 | N32@ND2 | 98.8 |
| | L455@O | Y33@HH | Y33@OH | 99.9 |
| H2 | S53@OG | R457@H | R457@N | 70.2 |
| | R457@O | S53@HG | S53@OG | 88.6 |
| | Y421@OH | G54@H | G54@N | 85.1 |
| | N460@OD1 | G55@H | G55@N | 9.0 |
| | T415@OG1 | S56@HG | S56@OG | 10.1 |
| | D420@OD1 | S56@HG | S56@OG | 97.4 |
| | D420@OD2 | S56@HG | S56@OG | 5.5 |
| | S56@OG | Y421@HH | Y421@OH | 6.8 |
| | S56@OG | N460@HD21 | N460@ND2 | 44.2 |
| H3 | N487@OD1 | R94@HH11 | R94@NH1 | 90.0 |
| | N487@OD1 | R94@HH22 | R94@NH2 | 99.6 |
| | G97@O | K417@HZ1 | K417@NZ | 26.3 |
| | G97@O | K417@HZ2 | K417@NZ | 34.6 |
| | G97@O | K417@HZ3 | K417@NZ | 34.2 |
| L1 | S28@O | Y505@HH | Y505@OH | 97.0 |
| | S30@OG | N501@HD21 | N501@ND2 | 5.0 |
| | N501@OD1 | S30@HG | S30@OG | 16.6 |
| L3 | G92@O | R403@HH12 | R403@NH1 | 18.3 |
| | G92@O | R403@HH21 | R403@NH2 | 12.9 |
| | D405@OD1 | S93@HG | S93@OG | 20.6 |
| | D405@OD2 | S93@HG | S93@OG | 13.0 |

**Table S3.** Hydrogen bond occupations of Fab CC12.3-D02 involved in SARS-CoV-2-RBD binding.

| CDRs | Acceptor | DonorH | Donor | Hydrogen bond occupancy (%) |
|---|---|---|---|---|
| - | S477@OG | Q1(H)@H1 | Q1(H)@N | 16.1 |
| | S477@OG | Q1(H)@H2 | Q1(H)@N | 17.0 |
| | S477@OG | Q1(H)@H3 | Q1(H)@N | 15.5 |
| | S67(L)@OG | Q498@HE22 | Q498@NE2 | 6.3 |
| H1 | G26@O | S477@H | S477@N | 5.9 |
| | G26@O | S477@HG | S477@OG | 16.3 |
| | K458@O | N31@HD22 | N31@ND2 | 5.7 |
| | N31@O | Y473@HH | Y473@OH | 29.3 |
| | T32@OG1 | Y473@HH | Y473@OH | 14.3 |
| | Y473@OH | A33@H | A33@N | 41.5 |
| H2 | D52@OD1 | K417@HZ1 | K417@NZ | 33.0 |
| | D52@OD1 | K417@HZ2 | K417@NZ | 14.0 |
| | D52@OD1 | K417@HZ3 | K417@NZ | 16.9 |
| | D52@OD1 | Y421@HH | Y421@OH | 62.8 |
| | D52@OD2 | K417@HZ1 | K417@NZ | 51.4 |
| | D52@OD2 | K417@HZ2 | K417@NZ | 20.4 |
| | D52@OD2 | K417@HZ3 | K417@NZ | 18.1 |
| | D52@OD2 | Y421@HH | Y421@OH | 69.5 |
| | Y421@OH | S54@H | S54@N | 71.4 |
| | S54@O | N460@HD21 | N460@ND2 | 28.9 |
| | D420@OD2 | S54@HG | S54@OG | 99.6 |
| | T415@OG1 | S56@HG | S56@OG | 69.9 |
| | D420@OD1 | S56@HG | S56@OG | 6.0 |
| | D420@OD2 | S56@HG | S56@OG | 19.1 |
| H3 | A475@O | R94@HH11 | R94@NH1 | 72.5 |
| | N487@OD1 | R94@HH12 | R94@NH1 | 98.4 |
| | N487@OD1 | R94@HH22 | R94@NH2 | 84.9 |
| | Y489@OH | R94@HH22 | R94@NH2 | 14.1 |
| | D96@OD1 | Y489@HH | Y489@OH | 15.5 |
| | D96@OD2 | Y489@HH | Y489@OH | 23.0 |
| | Y102@OH | N487@HD21 | N487@ND2 | 85.1 |
| L1 | Y505@OH | Q27@HE22 | Q27@NE2 | 11.1 |
| | D28@O | G502@H | G502@N | 83.6 |
| | D28@OD1 | T500@HG1 | T500@OG1 | 9.8 |
| | N501@OD1 | G30@H | G30@N | 17.6 |
| | Y495@O | Y31@HH | Y31@OH | 9.8 |
| L3 | A92@O | R403@HH12 | R403@NH1 | 87.8 |
| | A92@O | R403@HH21 | R403@NH2 | 83.3 |
| | E93@OE1 | Y505@HH | Y505@OH | 42.9 |
| | E93@OE2 | Y505@HH | Y505@OH | 52.3 |

**Table S4.** Hydrogen bond occupations of Fab CC12.3-D05 involved in SARS-CoV-2-RBD binding.

| CDRs | Acceptor | DonorH | Donor | Hydrogen bond occupancy (%) |
|---|---|---|---|---|
| - | S67(L)@OG | Q498@HE22 | Q498@NE2 | 11.5 |
| H1 | G26@O | N487@HD21 | N487@ND2 | 53.6 |
| | A475@O | N28@H | N28@N | 98.7 |
| | S30@OG | K458@HZ1 | K458@NZ | 5.3 |
| | S30@OG | K458@HZ2 | K458@NZ | 5.0 |
| | S30@OG | K458@HZ3 | K458@NZ | 5.1 |
| | A31@O | Y473@HH | Y473@OH | 95.3 |
| | D33@OD1 | K417@HZ2 | K417@NZ | 8.3 |
| | D33@OD2 | K417@HZ1 | K417@NZ | 9.3 |
| | D33@OD2 | K417@HZ2 | K417@NZ | 7.3 |
| | D33@OD2 | K417@HZ3 | K417@NZ | 7.3 |
| H2 | A53@O | K458@HZ1 | K458@NZ | 5.0 |
| | S54@OG | Y421@HH | Y421@OH | 5.2 |
| | Y421@OH | S54@HG | S54@OG | 13.8 |
| | N460@OD1 | S54@HG | S54@OG | 8.8 |
| | T415@OG1 | T56@HG1 | T56@OG1 | 6.3 |
| H3 | N487@OD1 | Y102@HH | Y102@OH | 80.8 |
| | N487@ND2 | Y102@HH | Y102@OH | 5.6 |
| | Y102@OH | N487@HD21 | N487@ND2 | 16.6 |
| L1 | D28@OD1 | N501@HD22 | N501@ND2 | 6.0 |
| | D28@OD1 | G502@H | G502@N | 52.6 |
| | D28@OD2 | N501@HD22 | N501@ND2 | 13.7 |
| | D28@OD2 | G502@H | G502@N | 87.3 |
| | Y31@OH | Q493@HE22 | Q493@NE2 | 13.3 |
| | S494@O | Y31@HH | Y31@OH | 77.5 |
| L3 | G92@O | R403@HH22 | R403@NH2 | 24.6 |
| | G92@O | Y505@HH | Y505@OH | 60.7 |
| | E93@OE1 | R403@HH12 | R403@NH1 | 61.8 |
| | E93@OE1 | R403@HH22 | R403@NH2 | 39.6 |
| | E93@OE1 | R408@HH12 | R408@NH1 | 90.4 |
| | E93@OE1 | R408@HH22 | R408@NH2 | 45.5 |
| | E93@OE2 | R403@HH12 | R403@NH1 | 36.7 |
| | E93@OE2 | R403@HH22 | R403@NH2 | 24.9 |
| | E93@OE2 | R408@HH12 | R408@NH1 | 86.4 |
| | E93@OE2 | R408@HH22 | R408@NH2 | 73.2 |

**Table S5.** Hydrogen bond occupations of Fab CC12.3-D08 involved in SARS-CoV-2-RBD binding.

| CDRs | Acceptor | DonorH | Donor | Hydrogen bond occupancy (%) |
|---|---|---|---|---|
| - | S67(L)@O | Q498@HE21 | Q498@NE2 | 92.0 |
| H1 | G26@O | N487@HD21 | N487@ND2 | 91.7 |
| | A475@O | T28@H | T28@N | 94.7 |
| | A31@O | Y473@HH | Y473@OH | 96.7 |
| H2 | Y421@OH | W52@HE1 | W52@NE1 | 5.2 |
| | L455@O | W52@HE1 | W52@NE1 | 91.9 |
| | Y421@OH | S54@H | S54@N | 21.1 |
| | D420@OD2 | S54@HG | S54@OG | 91.4 |
| | S54@O | N460@HD21 | N460@ND2 | 9.0 |
| | D420@OD1 | T56@HG1 | T56@OG1 | 14.8 |
| | D420@OD2 | T56@HG1 | T56@OG1 | 53.5 |
| H3 | N487@OD1 | R94@HH12 | R94@NH1 | 99.5 |
| | A475@O | R94@HH22 | R94@NH2 | 6.6 |
| | N487@OD1 | R94@HH22 | R94@NH2 | 99.5 |
| L1 | E27@OE1 | Y505@HH | Y505@OH | 5.3 |
| | D28@O | N501@HD22 | N501@ND2 | 51.8 |
| | D28@O | G502@H | G502@N | 98.0 |
| | D28@OD1 | Q498@HE22 | Q498@NE2 | 69.4 |
| | D28@OD1 | T500@HG1 | T500@OG1 | 57.1 |
| | D28@OD1 | N501@HD22 | N501@ND2 | 30.4 |
| | D28@OD2 | Q498@HE22 | Q498@NE2 | 75.4 |
| | D28@OD2 | T500@HG1 | T500@OG1 | 47.1 |
| | D28@OD2 | N501@HD22 | N501@ND2 | 35.1 |
| | G30@N | N501@HD21 | N501@ND2 | 6.8 |
| | Y31@OH | Q493@HE22 | Q493@NE2 | 30.8 |
| | S494@O | Y31@HH | Y31@OH | 75.7 |
| | S494@OG | Y31@HH | Y31@OH | 16.5 |
| | W32@NE1 | Q493@HE22 | Q493@NE2 | 10.5 |
| | S494@O | W32@HE1 | W32@NE1 | 70.0 |
| | Y495@O | W32@HE1 | W32@NE1 | 9.1 |
| L2 | D50@OD1 | Q493@HE21 | Q493@NE2 | 19.6 |
| | D50@OD2 | Q493@HE21 | Q493@HE21 | 44.1 |
| L3 | Y91@OH | Q493@HE21 | Q493@NE2 | 11.1 |
| | T92@O | R403@HH12 | R403@NH1 | 95.6 |
| | T92@O | R403@HH22 | R403@NH2 | 87.8 |
| | T92@OG1 | R403@HH22 | R403@NH2 | 63.2 |
| | D405@OD1 | K93@HZ1 | K93@NZ | 14.3 |
| | D405@OD1 | K93@HZ2 | K93@NZ | 15.3 |
| | D405@OD1 | K93@HZ3 | K93@NZ | 18.3 |
| | D405@OD2 | K93@HZ1 | K93@NZ | 18.8 |
| | D405@OD2 | K93@HZ2 | K93@NZ | 20.8 |
| | D405@OD2 | K93@HZ3 | K93@NZ | 23.5 |

**Table S6.** Pi interactions of Fabs CC12.3, CC12.3-D02, CC12.3-D05, and CC12.3-D08 involved in SARS-CoV-2-RBD binding.

| system | CDRs | residue that forms pi interaction with SARS-CoV-2-RBD | | | | |
|---|---|---|---|---|---|---|
| | | pi-pi | cation-pi | anion-pi | sigma-pi | alkyl-pi |
| CC12.3 | - | - | - | - | - | V2(H)---F486 |
| | H1 | Y33---F456 | - | - | - | - |
| | H2 | - | - | - | - | Y52---K417@CG |
| | H3 | F96---F456<br>F96---Y489 | R94@NH1---F486 | - | - | F99---L455 |
| | L1 | Y32---Y505 | Y32---R403@NH2 | - | - | - |
| | L2 | - | - | - | - | - |
| | L3 | - | - | - | - | - |
| CC12.3-D02 | H1 | - | - | - | - | A33---F456 |
| | H2 | - | - | - | - | A53---F456<br>A53---Y473 |
| | H3 | Y97---F456<br>Y102---F486 | Y97---K417@NZ | - | - | Y97---L455 |
| | L1 | Y31---Y449<br>W32---Y505 | W32---R403@NH2 | - | W32---Y505 | - |
| | L2 | - | - | E56@OE1---F486 | - | - |
| | L3 | - | - | - | - | A92---Y505 |
| CC12.3-D05 | - | - | - | - | - | V2(H)---F486 |
| | H1 | - | - | - | - | A31---Y473 |
| | H2 | W52---Y421 | W52---K417@NZ | - | - | W52---K417@CG |
| | H3 | Y102---F486 | - | - | - | I101---F486<br>I101---Y489 |
| | L1 | Y31---Y449<br>F32---Y505 | - | - | - | - |
| | L2 | - | - | - | - | - |
| | L3 | - | - | - | - | - |
| CC12.3-D08 | H1 | W33---F456<br>W33---Y489 | - | - | - | A31---Y473 |
| | H2 | W52---Y421<br>W52---F456 | W52---K417@NZ | - | W52---K417@HE2<br>W52---K417@HE3 | W52---K417@CG |
| | H3 | Y102---F486 | R94@NH1---F486 | - | - | - |
| | L1 | Y31---Y449<br>W32---Y453<br>W32---Y495 | W32---R403@NH2 | - | - | I29---Y505 |
| | L2 | - | - | - | - | - |
| | L3 | - | - | - | - | K93@CG---Y505 |
